# Prevalence of glucose-6-phosphate dehydrogenase (G6PD) deficiency in a malaria endemic region of Colombia: Implications for radical cure of *Plasmodium vivax*

**DOI:** 10.1371/journal.pgph.0007460

**Published:** 2026-11-11

**Authors:** Lina Zuluaga-Idárraga, Alexandra Rios-Orrego, Juan Esteban Martinez-López, Daniel Camilo Aguirre-Acevedo, Alberto Tobón-Castaño, Tatiana Lopera-Mesa, Iván Cardenas, Mario J. Olivera, Milton Cardozo-Cruz, Guillermo Gonzalvez, María Paz Ade, Roberto Montoya, Erika Venegas, Heidi Mihm, Sonia Herrera, Jaclyn Flewelling, Lawrence Barat, Xue Wu, Michelle S. Hsiang, Leopoldo Villegas

**Affiliations:** 1 Grupo Malaria, Facultad de Medicina, Universidad de Antioquia, Medellín, Antioquia, Colombia; 2 Grupo Epidemiología, Facultad Nacional de Salud Pública, Universidad de Antioquia, Medellín, Antioquia, Colombia; 3 Instituto de Investigaciones Médicas, Facultad de Medicina, Universidad de Antioquia, Medellín, Antioquia, Colombia; 4 Ministerio de Salud y Protección Social, Bogotá, Distrito Capital, Colombia; 5 Instituto Nacional de Salud, Bogotá, Distrito Capital, Colombia; 6 Pan American Health Organization (PAHO), Bogota, Distrito Capital, Colombia; 7 Pan American Health Organization (PAHO), Washington, District of Columbia, United States of America; 8 U.S. Agency for International Development (USAID), Bureau for Latin America and the Caribbean, Washington, District of Columbia, United States of America; 9 U.S. President’s Malaria Initiative (PMI) Impact Malaria Project, Washington, District of Columbia, United States of America; MRC Centre for Global Infectious Disease Analysis, Imperial College London, UNITED KINGDOM OF GREAT BRITAIN AND NORTHERN IRELAND

## Abstract

Glucose-6-phosphate dehydrogenase deficiency (G6PDd) has important implications for the treatment of *Plasmodium vivax*, the predominant malaria species in Latin America. The only drug class available for *P. vivax* radical cure, the 8-aminoquinolines, can induce hemolysis in individuals with G6PDd. However, G6PDd prevalence among subpopulations and its relationship with Plasmodium infection remain poorly characterized. In Quibdó, Colombia, an area co-endemic for *P. vivax* and *Plasmodium falciparum*, cross-sectional surveys were conducted in three groups: 1) the general population, 2) *Emberá dobida* indigenous communities, and 3) febrile outpatients. G6PD status was determined using a quantitative test (STANDARD™ G6PD Test, SD BIOSENSOR, South Korea), and Plasmodium infection was assessed by polymerase chain reaction. G6PD activity was classified as deficient (≤4.0 IU/g hemoglobin), intermediate (4.1–6.0 in females), or normal (≥6.1 in females, ≥ 4.1 in males). Among 1,267, 194, and 542 participants in groups 1, 2, and 3, respectively, G6PDd prevalence was 10.4% overall and 13.7% among males in the predominantly Afro-Colombian general population, with no cases among indigenous participants. Among febrile outpatients, G6PDd prevalence was 6.9% overall and 8.2% among males, with a comparable but slightly lower prevalence among those with confirmed malaria. Plasmodium infection prevalence was 17.8%, 40.7%, and 47.8%, respectively. *P. vivax* mono-infection predominated in community surveys (59.1% and 60.8%), whereas *P. falciparum* predominated among febrile outpatients (55.6%). After adjustment for sex, Afro-Colombian ethnicity, and prior malaria, G6PD-deficient individuals in the asymptomatic general population had a lower risk of Plasmodium infection, primarily *P. vivax* (aPR, 0.05; 95% CI, 0.01–0.18), and none had *P. vivax* infection. These findings highlight high G6PDd prevalence among Afro-Colombians and its absence among an indigenous population with the highest *P. vivax* burden, suggesting that G6PDd may confer partial protection against *P. vivax*. This information can inform prioritization of more effective radical cure for at-risk subpopulations.

## Introduction

Glucose-6-phosphate dehydrogenase deficiency (G6PDd) is the most common inherited metabolic condition, affecting more than 400 million individuals worldwide [1]. G6PD is a critical enzyme in red blood cells that that helps protect them against oxidative damage by regenerating nicotinamide adenine dinucleotide phosphate (NADPH) [1]. This X-linked condition primarily manifests as neonatal jaundice and acute hemolytic anemia, often triggered by oxidative stress resulting from infections, specific medications, or certain foods [1].

The geographic distribution of malaria closely overlaps with that of the most common G6PD gene variants (Mediterranean and African A- and A+) [1], suggesting potential co-evolutionary adaptation [2]. Although evidence remains inconsistent [3], it has been hypothesized that *Plasmodium*-infected red blood cells with reduced G6PD activity undergo oxidative stress that limits parasite replication, enhances splenic clearance, and reduces both parasitemia and disease severity [1].

G6PDd is of particular importance for the safety of radical cure of *Plasmodium vivax*, the predominant malaria species outside Africa. The 8-aminoquinolines, primaquine and tafenoquine, are the only available drugs that target the dormant liver hypnozoite stage of *P. vivax,* thereby preventing relapse [1,4,5]. However, these agents can cause potentially life-threatening hemolysis in individuals with G6PDd [1,4]*.* Ensuring the safe and effective use of 8-aminoquinolines is therefore essential, both for the treatment of symptomatic cases and potential mass drug administration in asymptomatic populations as part of malaria elimination strategies. Primaquine, the only widely available drug for radical cure over the past 70 years, requires a 7- or 14-day regimen and has variable efficacy depending on circulating parasite strains and adherence to treatment [6]. Although higher doses of primaquine (7 mg/kg) improve efficacy, they also increase the risk of hemolysis because of the dose-dependent interaction between 8-aminoquinolines and G6PDd [7]. Tafenoquine, a single-dose alternative shown to be non-inferior to low-dose primaquine (3.5 mg/kg), offers the advantage of complete adherence [8,9], but carries a higher potential for severe hemolysis in G6PD-deficient individuals because of its long half-life [10]. In contrast, the hemolytic effects of primaquine can be mitigated by discontinuing therapy if symptoms arise [10]. Another consideration is that malaria infection itself induces anemia and stimulates erythropoiesis. The resulting increase in young erythrocytes (which have higher G6PD activity than older cells) may partially reduce the hemolytic effects of 8-aminoquinolines, even in G6PD-deficient individuals [11]. Understanding the local epidemiology of G6PDd and its relationship with malaria is therefore essential to guide the safe use of 8-aminoquinoline-based therapies [12].

Globally, the prevalence of G6PDd varies considerably [13]. In Africa, prevalence often exceeds 10%, while in most parts of Latin America (LA), it is reported to be below 6%. However, higher prevalence has been observed in the Guianas, the Pacific coastal regions of Colombia and Ecuador, and along the Brazilian Atlantic coast (areas historically linked to African ancestry through the transatlantic slave trade) [13–19]. Regional data remain limited: many Latin American countries lack published surveys, and existing studies often focus on non-endemic areas or overlook variation in malaria status and genetic backgrounds. In Colombia, reported G6PDd prevalence ranges from 3.1% to 12.7%, with previous studies suggesting that Amerindian populations are largely unaffected [14,15].

In support of Colombia’s national malaria elimination goals [20], a deeper understanding of G6PDd heterogeneity across ethnic and demographic groups is needed. The primary objective of this study was to estimate the prevalence of G6PD phenotypes in Quibdó, a highly malaria-endemic region of Colombia, across three different populations: the largely Afro-Colombian general population, indigenous communities, and outpatients with suspected malaria. A secondary objective was to explore the relationship between G6PDd and the risk of *Plasmodium* infection.

## Methods

### Study site

The study was conducted in the municipality of Quibdó, the capital of Chocó Department, located in Colombia’s Pacific region along the Atrato River (Fig 1). According to the 2018 national census, Quibdó has an estimated population of 142,184 inhabitants [21]. The majority of the population is Afro-Colombian (91.4%), with approximately 90% residing in urban areas. The main economic activities include gold, silver, and platinum mining, and the municipality is affected by ongoing armed conflict.

**Fig 1:**
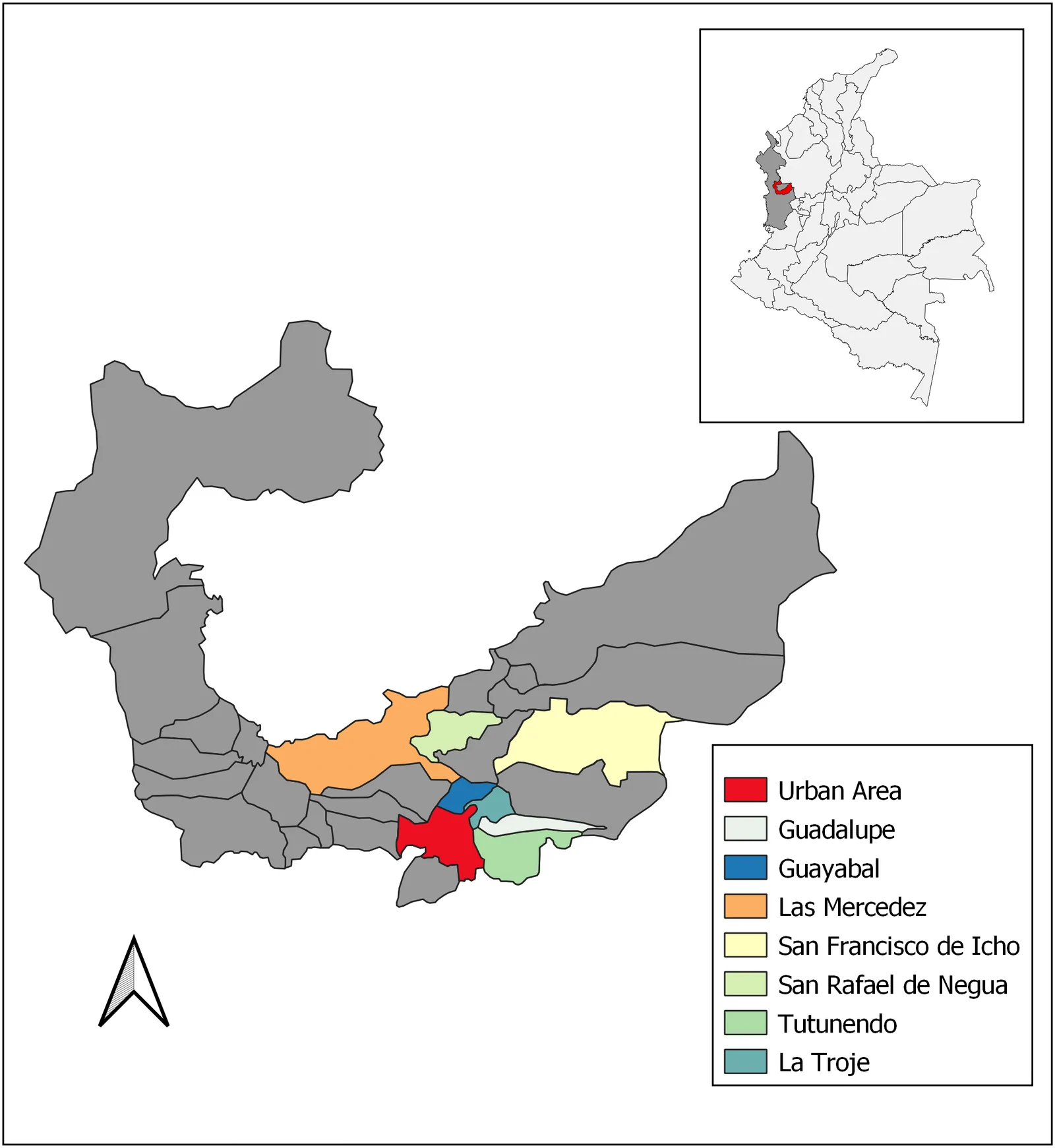
Study map. Quibdó municipality in the Pacific region of Colombia. The G6PDd prevalence survey among the general population was conducted in the urban area (red) and in seven of 27 rural communities. The figure was created using the open-source software QGIS (version 3.28). The base map shapefiles were obtained from official Colombian government sources; it was created using the municipal boundaries shapefile provided by the Colombian National Administrative Department of Statistics (DANE) (available at https://geoportal.dane.gov.co/servicios/descarga-y-metadatos/datos-geoestadisticos/).

In 2023, Chocó reported 37,160 malaria cases, accounting for 35.4% of Colombia’s total malaria burden. Of these, 18% occurred in Quibdó [22]. Malaria transmission occurs year-round in rural and peri-urban settings, with an incidence of 49.1 per 1,000 population. The species distribution was *P. falciparum* (70.8%), *P. vivax* (28.5%), and mixed (*P. falciparum/ P. vivax*) infection (0.6%).

### Study design and sample size

A cross-sectional survey was conducted from October 6 to December 4 2023 among three populations (referred to below as groups): (1) the largely Afro-Colombian general population in urban and rural areas of Quibdó; (2) indigenous communities in peri-urban areas; and (3) febrile outpatients with suspected malaria enrolled at two malaria diagnostic facilities affiliated with the Local Hospital Ismael Roldán Valencia (LHIRV). Inclusion criteria were age ≥ 5 years, absence of severe disease or acute systemic illness, and provision of informed consent. The Strengthening the Reporting of Observational Studies in Epidemiology (STROBE) guidelines were followed in this study.

#### General population (Group 1).

To estimate the prevalence of G6PDd in the general population, a sample size of 1,300 was calculated to measure an expected 6% G6PDd prevalence [17], 5% significance level, an approximately 2% margin of error, design effect of 2, and 20% refusal rate. Taking into consideration urban (90%; n = 1170) and rural (10%; n = 130), stratified random sampling of participants was conducted in localities with reported malaria cases in the first half of 2023 by LHIRV. Localities were selected based on their lower impact from armed conflict and the feasibility and safety of access (Fig 1).

Sampling of communities in the urban area was random and proportional to the population size of the neighborhoods with reported malaria cases (Fig 2). Community leaders of selected communities were invited to participate in a workshop regarding the study rationale and procedures, and were subsequently invited to facilitate study participation. When a community leader declined to participate or when access challenges related to armed conflict were identified, another neighborhood in the same commune (one administrative level above the neighborhood) was randomly selected to replace it. Community leaders provided lists of households, from which 1,170 households were randomly selected (assuming one participant per household) and visited to collect demographic information on inhabitants (sex, age, and availability to participate in the survey). When the visit was not possible, another household from the same neighborhood was randomly selected to replace it. In selected households, one individual meeting the inclusion criteria was randomly selected and invited to participate in the survey. If a household had to be removed from the list of selected households (no inhabitants were available during sample collection, or the family moved to another location after the initial visit), an additional individual per household was included.

**Fig 2:**
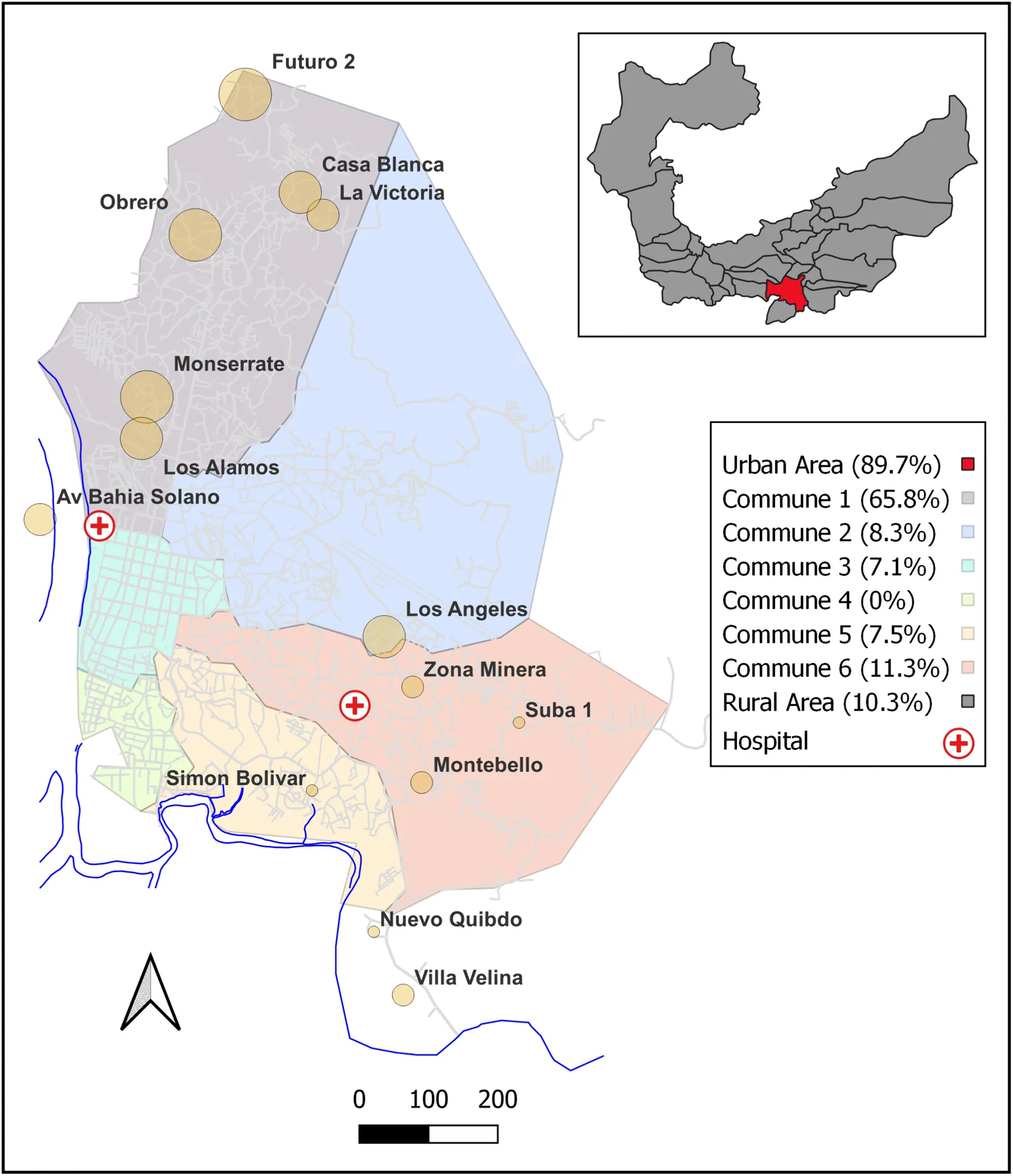
Sampling in the urban area. Sampling was proportional to the population size of neighborhoods reporting malaria cases; the percentages of samples from each locality are shown. The figure was created using the open-source software QGIS (version 3.28). The base map shapefiles were obtained from official Colombian government sources; it was created using a map available on the official website of the Municipality of Quibdó (available at https://www.quibdo-choco.gov.co/MiMunicipio/Paginas/Informacion-del-Municipio.aspx).

In the rural area (Fig 1), community health workers provided a list of all households and inhabitants. One individual per household meeting the inclusion criteria was randomly selected and invited to participate in the survey. An additional individual per household was included if a participant had to be replaced and no additional households were available for inclusion (a small number of households per locality).

#### Indigenous population (Group 2).

A sample size of 211 participants was calculated to measure an expected G6PDd prevalence of 1% [15] at a 5% significance level, and assuming 1.34% margin of error, design effect of 1, and no anticipated refusal. The study staff contacted the legal representative of one of the largest indigenous associations in Quibdó. Ten indigenous communities of the *Emberá dobida* ethnic group belonged to this association, comprising approximately 468 inhabitants of peri-urban neighborhoods of Quibdó. A meeting was held with indigenous governors to explain the study rationale and procedures, and to request preliminary approval to recruit participants. Eight communities agreed to participate and provided census data. The participants were randomly selected from the sampling frame of individuals who met the inclusion criteria.

#### Outpatients with suspected malaria (Group 3).

The sample size of 542 was recruited to measure an expected G6PDd prevalence of 6% [17] at 5% significance level, and assuming approximately 2% error, design effect of 1, and 12% refusal rate. Two malaria diagnostic posts affiliated with the Local Hospital (LHIRV) were selected; one was located within the hospital in the urban area, and the other in a rural area (Tutunendo locality). In 2023, approximately 40% of the reported malaria cases in Quibdó originated from these facilities. Febrile outpatients with suspected malaria who met the inclusion criteria were invited to participate. Convenience sampling was used, with participants enrolled consecutively until the required sample size was reached.

### Data and sample collection

A structured questionnaire was administered to collect demographic, epidemiological, and clinical information, including a history of malaria and signs and symptoms consistent with adverse events (mainly hemolysis) during the last self-reported malaria episode. Capillary blood was collected by finger prick for rapid G6PD activity testing. An additional 250 μL of blood was collected and preserved in DNA/RNA Shield™ (1:1 ratio) at ambient temperature, then transported to the Malaria Group Unit at the Universidad de Antioquia (Medellin) for processing within 15 days.

### Laboratory procedures

#### G6PD activity testing.

STANDARD™ G6PD Test (SD BIOSENSOR, South Korea) was performed using 10µL of non-anticoagulated blood, collected by finger prick as per the manufacturer’s instructions. The quantitative test result was classified according to the manufacturer’s recommendations [23]: ≤ 4.0 IU/g hemoglobin (Hb) deficient; 4.1–6.0 IU/g Hb intermediate in females, ≥ 4.1 IU/g Hb normal in males, and ≥ 6.1 IU/g Hb normal in females. For participants showing deficient or intermediate results, the test was repeated and the average of both measurements was used for the analyses. The study staff provided verbal counseling for deficient and intermediate participants regarding the risk of hemolysis with the use of primaquine (tafenoquine is not currently available or used in Colombia). For the STANDARD™ G6PD Test, the valid measurement range for G6PD activity is 0–20 IU/g Hb and can be calculated when the Hb concentration is between 4 and 25 g/dL. However, according to the manufacturer’s instructions, although the analyzer may generate a G6PD result when the Hb concentration is < 7 g/dL, these results should not be interpreted. Therefore, in this study, G6PD results from participants with Hb < 7 g/dL were excluded from all analyses involving G6PD level or status classification. These participants were referred for confirmation of anemia status and medical evaluation.

#### Molecular detection of *Plasmodium* spp.

Automated DNA extraction was carried out using Thermo Fisher Scientific - MX® (KingFisher instruments) according to the manufacturer’s instructions using 200 µL of the whole-blood and DNA/RNA Shield™ mixture. A 5 µL aliquot of extracted DNA was used as a template to amplify a region of the multicopy 18S rRNA gene of the genus *Plasmodium* by qPCR [24]. Reactions were performed using Platinum Quantitative PCR superMix-UDG TaqMan Universal Master Mix (Invitrogen Thermo Scientific) on an Agilent AriaMx Real-Time PCR System. A positive result was determined according to standardized parameters of cycle threshold (Ct), ΔR last (RFU; final fluorescence units) and amplification curve. The limit of detection was 1 parasite/µL.

*Plasmodium-*positive samples were processed for species-specific detection. First, species-specific testing for *P. falciparum* and *P. vivax* was conducted using two independent qPCR assays [25] and 5x Hot FIREPol EvaGreen qPCR Supermix. A positive result was determined according to standardized parameters of Ct, ΔR last, and melting temperature (Tm). The limit of detection was five parasites/µL. Samples that did not amplify in the *P. falciparum* or *P. vivax* species-specific testing were additionally processed by nested PCR. Samples that remained negative were subsequently tested for *Plasmodium malariae* by nested PCR [25]. Laboratory technicians were blinded to clinical and epidemiological metadata.

#### Microscopy.

Malaria diagnosis by microscopy was performed in Group 3 participants with suspected malaria. Thick and thin blood films were prepared from capillary blood samples and stained with Giemsa. Trained microscopists from local hospital staff examined the slides and administered treatment according to the guidelines of the Colombia’s Ministry of Health guidelines. Microscopy results were recorded on a study data collection form.

### Data management and statistical analysis

Demographic, clinical information, and as well as laboratory results collected in the field, were recorded on paper and subsequently entered into electronic forms after quality review by a field supervisor and a senior expert. Incoming data were reviewed daily by the data manager based in Medellín. Molecular testing results were entered separately into the study database separately.

Data verification and validation were conducted through manual review of 10% of the questionnaires (200 of 2,003) by comparing them with the corresponding electronic records to assess consistency, accuracy, and completeness. Data were then cleaned and analyzed using R version 4.3.0, RStudio version 2023.03.1 + 446 and QGIS version 3.28.2-Firenze.

A descriptive analysis was performed for the participant characteristics overall and by study group. When the G6PD test was repeated, the intraclass correlation coefficient (ICC) and Bland-Altman plots were used to evaluate agreement in G6PD activity and hemoglobin levels between the two tests for each participant (n = 246). The mean of the two measurements was used for analyses when both measurements fell within the Bland–Altman limits of agreement and G6PD classification did not change (n = 231). For the remaining participants, results were individually reviewed to rule out potential technical errors and determine which of the two measurements would be used for analyses (n = 15). The prevalence of G6PDd and *Plasmodium* infection was estimated accounting for the probability of selection under the sampling design. To account for variability in the prevalence of G6PDd in the study populations, G6PD classifications were also made using the adjusted median within each group [26].

The associations between sex, ethnicity, previous malaria diagnosis, and current Plasmodium infection, and G6PD status (deficient versus normal or intermediate) [27–29] were assessed using a robust Poisson regression method to estimate prevalence ratios [30]. Group 2 was not included in this analysis because no cases of G6PDd were identified among participants from the indigenous populations. A generalized estimating equations (GEE) model was used for Group 1 to account for sampling weights and the correlation between measurements at the household level (25.9% of households had two participants). A generalized linear model (GLM) was used for Group 3 to explore the association between the logarithm of G6PD activity (IU/g Hb) and *Plasmodium* infection.

### Ethical considerations

Ethical approval was obtained from the institutional review boards: Universidad de Antioquia (Record No. 055), Instituto Nacional de Salud (CEMIN 21-2022), University of California, San Francisco (22–36838; Ref. No. 350141), and the Pan American Health Organization (PAHOERC.0597.02). Written informed consent was obtained from adult participants, while parental or guardian consent and child assent were obtained for participants aged 5–17 years. Molecular test results were not used for clinical management because the analyses were conducted three months post-fieldwork. Informed consent forms were provided in Spanish, with translation support from indigenous community interpreterswhen needed.

### Inclusivity in global research

Additional information regarding the ethical, cultural, and scientific considerations specific to inclusivity in global research is included in the Supporting Information (S1 Checklist).

## Results

### Descriptive characteristics of the study populations

In urban areas, 35 neighborhoods across five of the six communes met the selection criteria; whereas rural areas, 7 of 27 neighborhoods met the selection criteria. A total of 2,003 participants were enrolled: 1,267 from the general population, 194 from the *Emberá dobida* indigenous population, and 542 outpatients (Fig 3).

**Fig 3:**
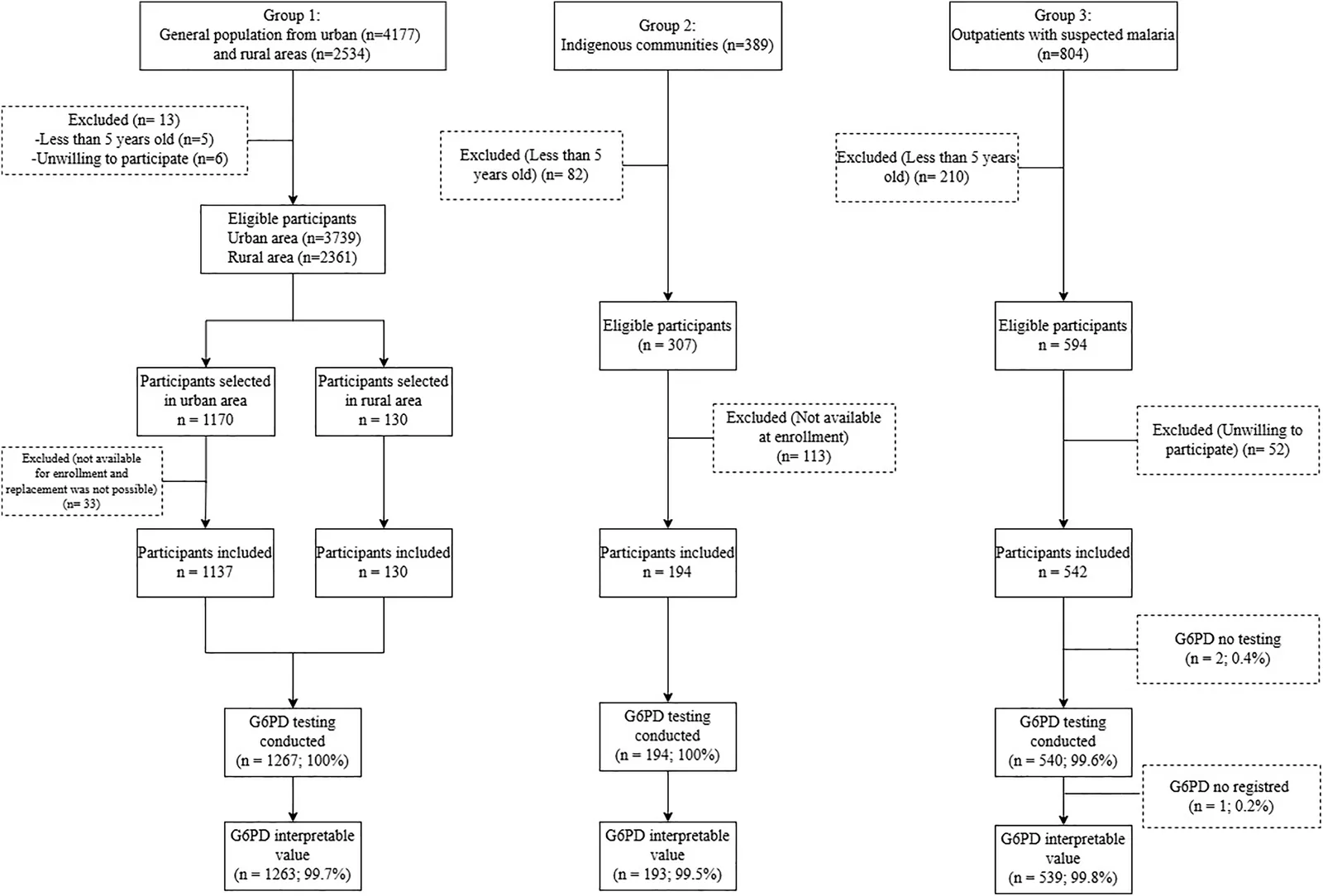
Flowchart of enrollment and glucose-6-phosphate dehydrogenase (G6PD) testing.

Table 1 summarizes participants’ epidemiological characteristics, malaria history, and malaria testing results. A higher proportion of females was recruited from the general population. Participants in the general population and outpatient groups were, on average, older than those in the indigenous group. Afro-Colombians comprised nearly the entire general population (96.8%) and 73.4% of outpatients; while indigenous participants represented 19.2% of outpatients.

**Table 1:** Characteristics of study participants.

	General population (Group 1)	Indigenous (Group 2)	Outpatients (Group 3)
	(n = 1,267)	(n = 194)	(n = 542)
**Female, n (%)**	814 (64.2%)	104 (53.6%)	271 (50.0%)
**Age, years, mean (SD)**	37.3 (21.3)	24.0 (16.1)	34.7 (18.4)
**Age group, n (%)**			
5–11 years	124 (9.8%)	53 (27.3%)	47 (8.7%)
12–14 years≥	80 (6.3%)	21 (10.8%)	31 (5.7%)
≥ 15 years	1,052 (83.0%)	118 (60.8%)	449 (82.8%)
**Ethnic group (self-report), n (%)**			
Indigenous	9 (0.7%)	192 (99.0%)	104 (19.2%)
Afro-Colombian	1,227 (96.8%)	0 (0%)	398 (73.4%)
Other	22 (1.7%)	2 (1.0%)	37 (6.8%)
Consanguinity between parents*, n (%)	1 (0.1%)	2 (1.0%)	0 (0%)
**Years of residence in the study location, mean (SD)**	27.9 (20.0)	11.5 (12.7)	17.3 (16.8)
**Sleep at least one night outside the residence in the last three months; n (%)**	69 (5.4%)	2 (1.0%)	203 (37.5%)
Malaria transmission in the place visited; n (%)^₤^	39 (56.5%)	1 (50.0%)	182 (89.6%)
**Previous self-reported malaria episodes; n (%)**	545 (43.0%)	121 (62.4%)	409 (75.5%)
Lifetime malaria episodes; mean (SD) ^€^	4.1 (4.5)	2.9 (2.8)	4.9 (5.4)
Time since last episode; n (%)^€^			
Less than six months	63 (11.6%)	42 (34.7%)	207 (50.6%)
More than six months	426 (78.2%)	75 (62.0%)	183 (44.7%)
Does not remember	49 (8.9%)	2 (1.6%)	10 (2.4%)
**Fever in the last three days; n (%)**	4 (0.3%)	2 (1.0%)	464 (85.6%)
**Hemoglobin (Hb, g/dL); mean (SD)**	13.4 (2.2)	12.6 (2.1)	13.4 (2.2)
**Plasmodium infection by PCR**			
Negative	1,049 (82.8%)	114 (58.8%)	283 (52.2%)
*Plasmodium* positive	193 (15.2%)	79 (40.7%)	259 (47.8%)
*P. vivax*‡	114 (59.1%)	48 (60.8%)	100 (38.6%)
*P. falciparum*‡	16 (8.3%)	15 (19%)	144 (55.6%)
Mixed *P. falciparum*/*P. vivax*‡	2 (1.0%)	3 (3.8%)	13 (5.0%)
Species not determined‡	61 (31.6%)	13 (16.4%)	2 (0.8%)
Not processed	25 (2.0%)	1 (0.5%)	0 (0%)

Frequency of demographic, epidemiological, and clinical characteristics of study participants.

* Genetic relatedness is relevant toG6PDd; parental consanguinity was determined based on participants’ self-reported parental relatedness.

**^₤^** Percentage calculated among participants who slept at least one night away from their residence during the previous three months.

**^€^** Percentage calculated among participants with a previous diagnosis of malaria.

‡ Percentage calculated among participants with any *Plasmodium* infection.

SD: Standard deviation.

A previous self-reported malaria diagnosis was most common among outpatients (75.5%), followed by indigenous participants (62.4%) and the general population (43.0%); a similar pattern was observed for self-reported malaria within the previous six months. The prevalence of *Plasmodium* spp*.* infection was 17.8% in the general population, 40.7% in the indigenous population, and 47.8% among outpatients with suspected malaria (Table A in S1 Appendix). All infected participants identified through the community-based surveys (general and indigenous populations) were asymptomatic at enrollment, and <1% reported fever during the preceding three days. Among outpatients with malaria symptoms, 55.6% (144/259) of infections were due to *P. falciparum*. In contrast*, P. vivax* predominated among asymptomatic infections in the general (59.1%, 114/193) and indigenous (60.8%, 48/79) populations (Table A in S1 Appendix).

### G6PD test results

Overall, 12.3% (246/2,003) of participants underwent repeat testing because of an initial deficient or intermediate result. Repeat measurements of G6PD enzymatic activity and hemoglobin (Hb) showed high agreement (Fig A and Fig B in S1 Appendix). No G6PD activity values fell outside the manufacturer’s specified measurement range. One Hb value was outside the device’s measurement range (4–25 g/dL), and 8 participants (0.4%) had Hb < 7 g/dL.

Quantitative G6PD activity by study population, including a subgroup of outpatients restricted to Afro-Colombians is presented in Table 2 and Fig C in S1 Appendix. Distributions by sex and population are shown in Fig 4. The distributions in all populations, including Afro-Colombians, showed a left-sided shoulder consistent with G6PDd. Median G6PD activity was lower in males than in females and was lowest among males in the predominantly Afro-Colombian general population and among Afro-Colombian outpatients.

**Table 2:** G6PD enzymatic activity (IU/g/Hb) and prevalence of G6PD status.

	General population	Indigenous	Outpatients	Outpatients restricted to Afro-Colombians
	(Group 1) ^₤^	(Group 2)	(Group 3)	(subgroup of Group 3)
	n = 1263	n = 193	n = 539	n = 397
**G6PD activity UI/g Hb; Median [IQR]**				
**Overall**	7.10 [6.00, 8.10]	9.70 [8.40, 11.10]	8.10 [6.70, 9.30]	7.80 [6.45, 9.00]
Overall adjusted*	7.20 [6.20, 8.20]	9.80 [8.50, 11.10]	8.20 [7.10, 9.40]	7.90 [6.60, 9.00]
**Sex**				
Female	6.90 [6.10, 8.20]	9.80 [8.45, 11.20]	8.30 [7.10, 9.40]	8.00 [6.88, 9.10]
Male	7.30 [5.70, 8.10]	9.50 [8.40, 10.67]	7.90 [6.40, 9.20]	7.40 [6.00, 8.65]
**Ethnicity**				
Afro-Colombians	7.10 [6.00, 8.10]		7.80 [6.45, 9.00]	7.80 [6.45, 9.00]
Indigenous		9.70 [8.40, 11.10]	9.00 [8.10, 10.17]	
Other	8.60 [7.30, 9.80]		8.45 [6.90, 9.75]	
**Prevalence of G6PD status, % [95% CI]**				
**Deficient**	10.4% [8.2–13.0]	^€^	6.9% [5.0–9.5]	9.4% [6.8–12.8]
Female	8.2% [5.9–11.3]	^€^	5.6% [3.3–9.3]	7.5% [4.4–12.3]
Male	13.7% [9.7–18.9]	^€^	8.2% [5.3–12.3]	11.3% [7.4–16.8]
**Intermediate (female)**	9.1% [7.2–11.5]	1.0% [0.2–4.1]	4.1% [2.7–6.2]	4.8% [3.0–7.5]
Prevalence of G6PD status, % of activity classification*; % [95% CI]				
< 30%	2.0% [1.1–3.5]	^€^	3.5% [2.2–5.6]	4.8% [3.0–7.5]
30-69%	14.8% [12.2–17.9]	4.1% [1.9–8.3]	9.7% [7.4–12.6]	12.4% [9.4–16.2]
≥ 70%	83.2% [80.0–86.0]	95.9% [91.7–98.1]	86.8% [83.5–89.4]	82.8% [78.6–86.3]

G6PD enzymatic activity value (IU/g/Hb) and prevalence of G6PD status, overall and by subgroup.

* An “adjusted median” was calculated by excluding males with G6PD activity ≤10% of the male median. The “adjusted median” was considered 100% G6PD activity, and non-sex-specific cut-off levels were determined according to WHO guidelines [26].

^**₤**^ Estimates account for sample weights.

€ Value not calculated because did not identify G6PDd cases in this group.

IQR: Interquartile Range.

**Fig 4:**
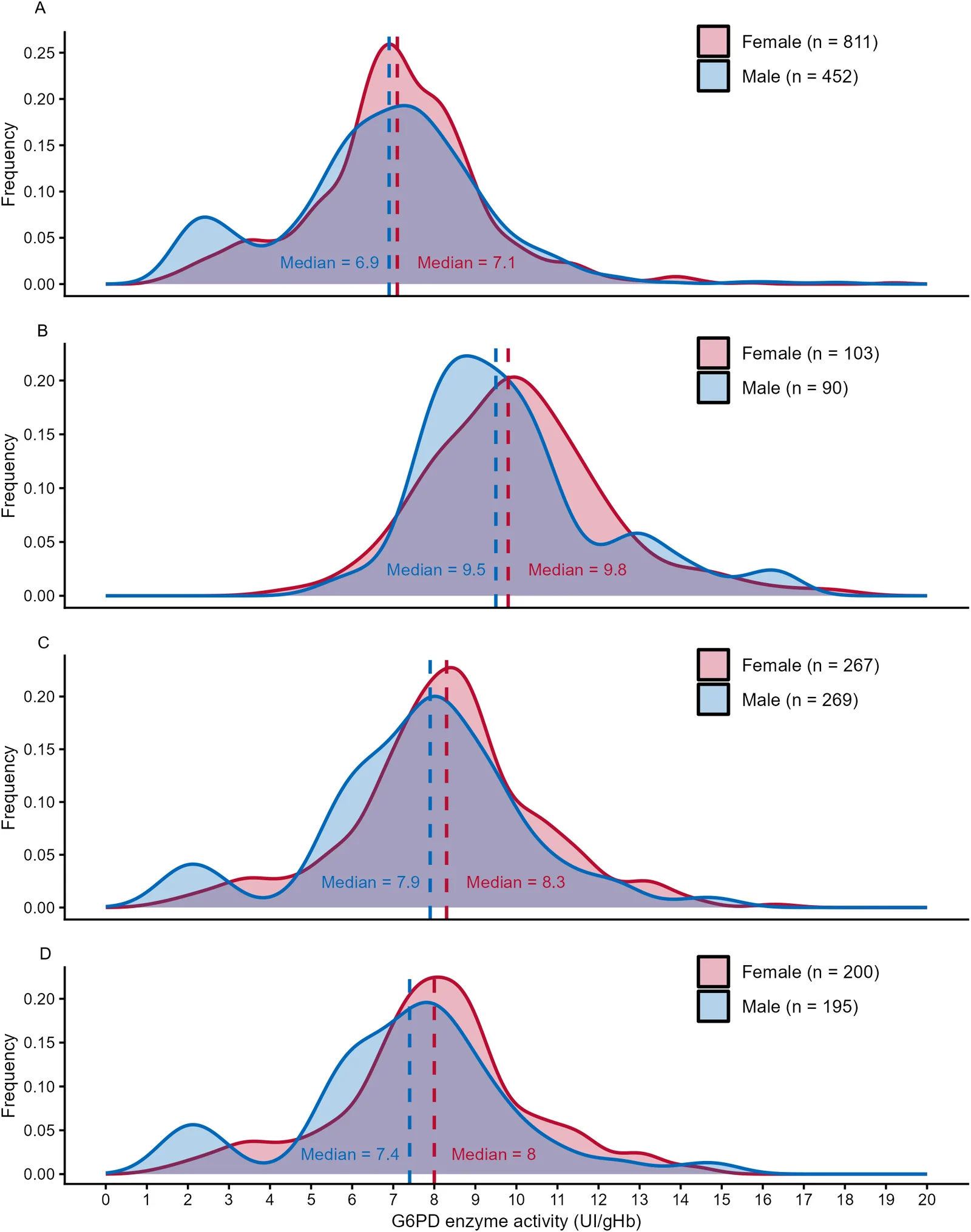
Distribution of G6PD activity by sex. (A) general population (Group 1), (B) indigenous communities (Group 2), (C) outpatients (Group 3), and (D) outpatients restricted to Afro-Colombians. Female values in pink, male values in blue; medians shown as dotted vertical lines.

Across study populations, G6PDd prevalence was highest in the predominantly Afro-Colombian general population (10.4%), followed by outpatients (6.9%); no G6PDd cases were detected in the indigenous communities (Table 2). Given the high prevalence among Afro-Colombians in the general population, G6PDd prevalence among outpatients restricted to Afro-Colombians is also reported. The highest prevalence among male was observed in the general population (13.7%) and among Afro-Colombian outpatients (11.3%).

Intermediate status among females was 9.1% in the predominantly Afro-Colombian general population, 1.0% in the indigenous population, and 4.8% among outpatients restricted to Afro-Colombians (Table 2).

### Self-reported prior history of G6PDd, hemolytic anemia, and adverse events with primaquine

Self-reported history of G6PDd, hemolytic anemia, and adverse events following primaquine intake are presented in Table B in S1 Appendix. The frequencies of prior G6PDd diagnosis or hemolytic events (including hospitalization or blood transfusion) were similar across participants with deficient, intermediate, and normal G6PD status. Overall, 25.8% (513/1,992) reported having taken primaquine during their most recent malaria episode; 56 participants reported adverse events, most commonly dizziness or vomiting. Reported adverse events were less frequent among G6PD-deficient participants (7%) than among those with intermediate (20%) or normal (12%) G6PD status.

### Association between G6PDd and risk of current malaria infection

Across all study populations, mean G6PD activity levels were higher among participants with a current *Plasmodium* infection than among those without infection (Fig 5A). A similar pattern was observed for *P. vivax* compared with no infection (Fig 5B). In the general population, the trend was reversed for *P. falciparum*, although the number of *P. falciparum* infections in this group was small (Fig 5B).

**Fig 5:**
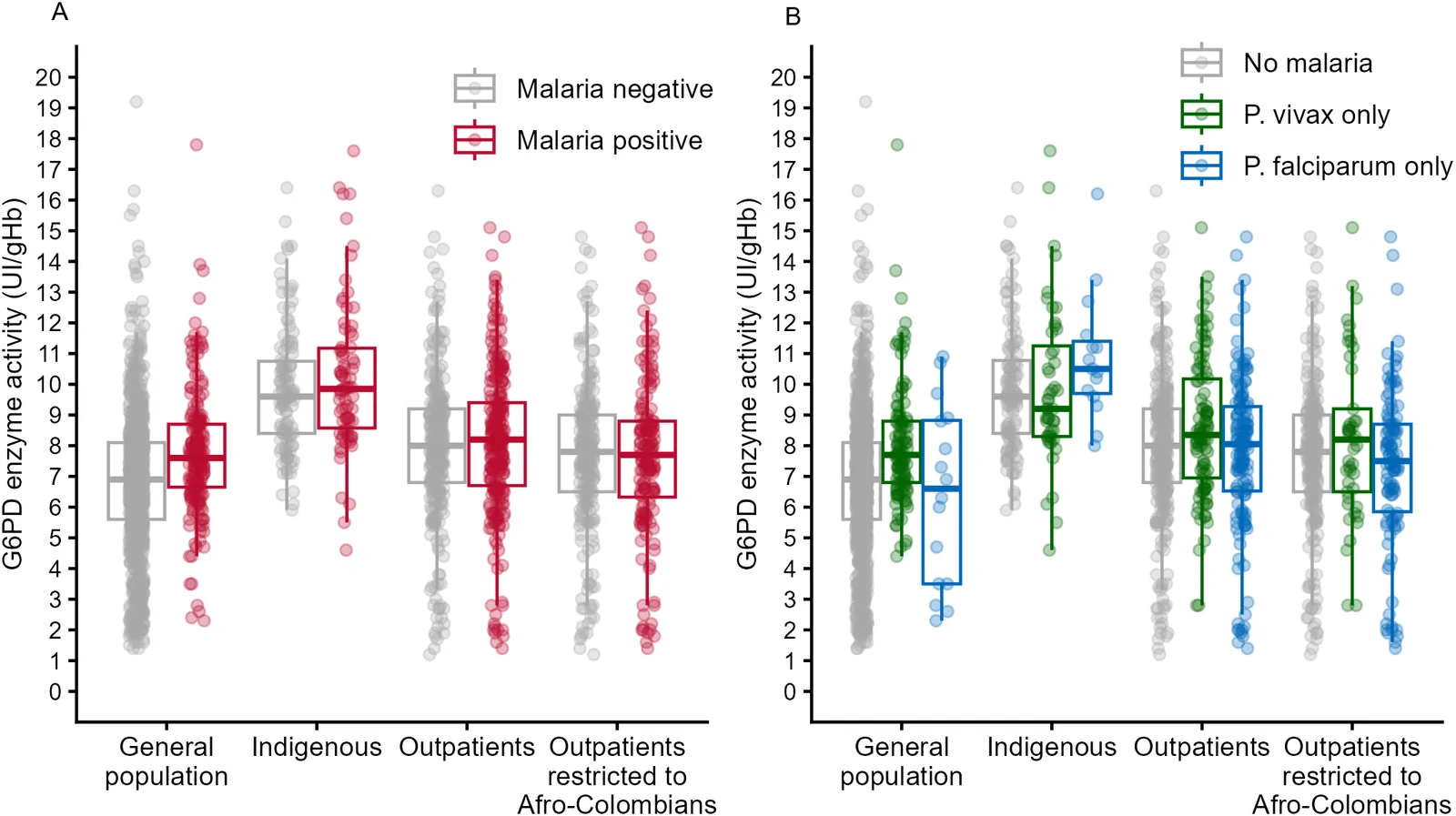
G6PD activity by current Plasmodium infection across study populations. **(A)** Any Plasmodium infection versus none. **(B)** By species. Median and IQR are shown; whiskers extend to 1.5 × IQR.

In the general population, the prevalence of any *Plasmodium* infection and of *P. vivax* was lower among G6PD-deficient participants than among those with normal G6PD activity (Fig 6 and Table C in S1 Appendix). Notably, no *P. vivax* infections were detected among individuals with G6PDd. Conversely, *P. falciparum* prevalence was higher in G6PD-deficient individuals and lower among females with intermediate G6PD activity than in those with normal activity (Fig 6). The indigenous population was excluded from these analyses because no G6PDd cases were identified.

**Fig 6:**
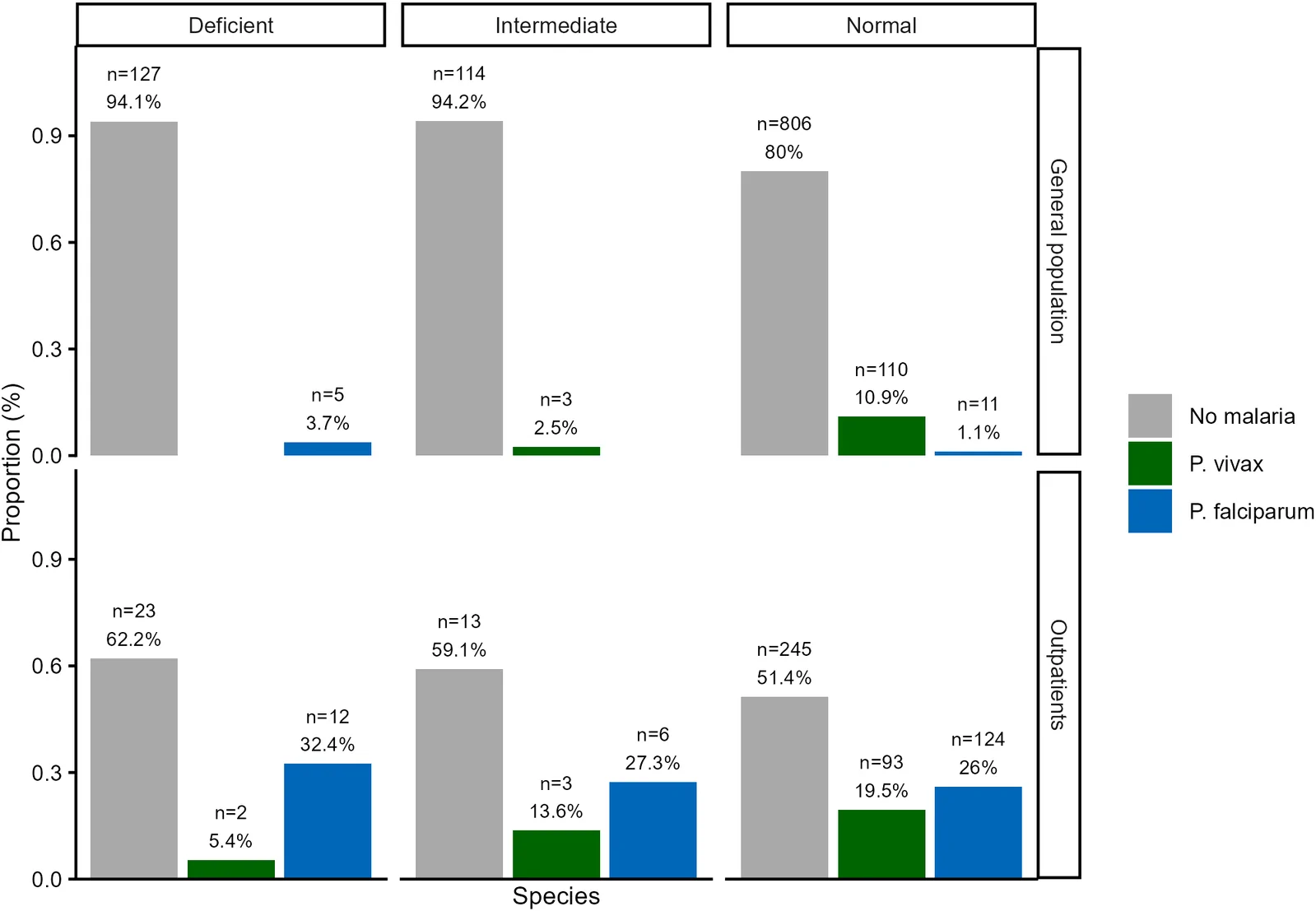
Prevalence of *Plasmodium* infection by G6PDd status group. Prevalence of *Plasmodium* infection according to G6PDd status (deficient, intermediate, and normal) and study group. Group 2 not included due to lack of G6PDd in indigenous communities.

In the study groups with G6PDd (general population and outpatients), the associations between G6PDd status or G6PD activity level and current *Plasmodium* infection were assessed. In the general population where *P. vivax* was the predominant species, G6PDd was associated with a lower risk of Plasmodium infection compared with normal/intermediate G6PD status as the reference category, both in unadjusted and adjusted models controlling for ethnic group, sex, and history of malaria (PR 0.05; 95% CI 0.01 – 0.23; aPR 0.05; 95% CI 0.01 – 0.18) (Table 3). When G6PD activity was analyzed as a continuous variable in linear models (Table C in S1 Appendix), *Plasmodium* infection was associated with a 28% (95% CI 19% –37%) increase in G6PD activity in the general population, after adjusting for ethnic group, sex, and history of malaria (Table D in S1 Appendix). These associations were not observed in outpatients where *P. falciparum* was the predominant species: G6PD status as a binary variable or continuous variable was not associated with current *Plasmodium* infection (Table 3, Table C and Table D in S1 Appendix).

**Table 3:** Prevalence ratios (PRs) for associations between sex, ethnicity, malaria, and G6PD deficiency (G6PDd) by group.

	General population				Outpatients with suspected malaria				Outpatients with suspected malaria restricted to Afro-Colombians			
	(Group 1, n = 1263)^£^				(Group 3, n = 539) ^¥^				(Group 3, n = 397) ^¥^			
	Crude		Adjusted*		Crude		Adjusted*		Crude		Adjusted*	
Factors	PR	CI 95%	aPR	CI 95%	PR	CI 95%	aPR	CI 95%	PR	CI 95%	aPR	CI 95%
**Afro-Colombian**	0.71	0.20–2.52	0.85	0.23–3.19	^€^		^€^					
**Male**	1.76	1.12–2.75	2.23	1.45–3.44	1.46	0.78–2.80	1.51	0.88–2.61	1.50	0.81–2.86	1.51	0.81–2.90
**Prior reported history of malaria**	1.67	1.05–2.67	1.72	1.11–2.66	0.86	0.44–1.82	0.93	0.46–1.99	0.96	0.50–2.01	0.96	0.48–2.04
**Current *Plasmodium* infection, any species**	0.05	0.01–0.23	0.05	0.01–0.18	0.67	0.35–1.26	0.65	0.33–1.25	0.88	0.45–1.63	0.85	0.44–1.63
*P. falciparum*	0.98	0.38–2.54	0.75	0.26–2.17	1.03	0.51–1.99	1.00	0.48–1.99	1.14	0.57–2.17	1.10	0.54–2.16
*P. vivax*	^€^	^€^	^€^	^€^	0.25	0.04–0.81	0.24	0.04–0.80	0.45	0.08–1.45	0.45	0.08–1.47
Others	0.04	0.01–0.25	0.04	0.01–0.16	^€^	^€^	^€^	^€^	^€^	^€^	^€^	^€^

Prevalence ratios (PR) for associations between sex, ethnicity, malaria, and G6PD deficiency (G6PDd). Results shown by group, with Indigenous population not included due to lack of G6PDd.

£ Estimates by generalized structural equation model (GEE).

¥ Estimates by generalized linear model (GLM).

€ Value were not estimated because some categories did not contain G6PDd cases.

*Adjusted by all variables in the table.

Species-specific analyses showed that no participant in the general population with *P. vivax* infection had G6PDd; therefore, it was not possible to estimate the magnitude of the association (Table 3). However, *P. vivax* infection was associated with a 29% increase in G6PD activity (95% CI 19–40%) in the general population (Table D in S1 Appendix). In outpatients, G6PDd was associated with risk of *P. vivax* infection (PR 0.25; 95% CI 0.04 – 0.81 and aPR 0.24; 95% CI 0.04 – 0.80). Consistent with these findings *P. vivax* infection was associated with a 12% increase in G6PD activity (95% CI 3–23%) compared with no infection (Table D in S1 Appendix). In both the general population and outpatients, *P. falciparum* infection was not associated with G6PD as a binary variable or continuous variable (Table 3, Table C and Table D in S1 Appendix).

## Discussion

This study provides the first population-based estimates of G6PDd among both Afro-Colombian and Indigenous populations in a malaria-endemic region of Colombia. Three main findings emerged. First, G6PDd prevalence was high among the Afro-Colombian population but was not detected in the Indigenous communities included in this survey, highlighting substantial heterogeneity in G6PDd prevalence across populations with different ancestry. Second, G6PDd was associated with a lower prevalence of *P. vivax* infection, supporting the hypothesis that reduced G6PD activity may confer partial protection against this parasite. Finally, these findings provide important epidemiological evidence to inform the implementation of new radical cure strategies, particularly in settings where the prevalence of G6PDd and the burden of *P. vivax* vary substantially acrosssub-populations.

The high prevalence of G6PDd observed among Afro-Colombians in Quibdó is consistent with the known distribution of G6PD variants in populations of African ancestry. Similar prevalence estimates have been reported in Africa and among Afro-descendant populations in Colombia and other parts of Latin America and the Caribbean [13,17,19,31,32]. Prevalence estimates from populations with a greater degree of genetic admixture, such as those in the Brazilian Amazon, have generally been lower, highlighting the influence of population ancestry on the distribution of G6PDd [16]. Comparisons across studies should, however, be interpreted with caution because differences in diagnostic methods and activity thresholds used to define G6PDd may contribute to variability in reported prevalence.

This study provides the first estimate of G6PDd prevalence in an Indigenous population in Colombia. G6PDd has been suggested to be uncommon among Indigenous and Indigenous-European admixed populations in Latin America [33], but available evidence remains scarce [31]. A study among Indigenous populations in Ecuadorfound a markedly lower frequency of the G6PD A− variant than that observed among Afro-descendant populations from the same region [15]. The absence of G6PDd in the *Emberá dobida* population may reflect the limited genetic admixture that has been described in this ethnic group. The marked contrast with the Afro-Colombian population living in the same municipality highlights the substantial heterogeneity in G6PDd prevalence that may exist within relatively small geographical areas. These findings suggest that estimates of G6PDd should not be extrapolated solely on the basis of geographic proximity and reinforce the importance of considering population ancestry when planning G6PD testing strategies.

G6PDd was associated with an absence of *P. vivax* infection among the largely asymptomatic general populations (0 infections among the 132 G6PD deficient participants), and although not statistically significant, the association was in the same direction for outpatients. *P. vivax* infection was also associated with 29% and 12% higher G6PD activity in the general population and outpatients, respectively. While, *P. vivax* bone marrow suppression might counterbalance the reticulocytosis associated with a malaria episode, a nonetheless plausible biological explanation for the lower magnitude associations measured in outpatients versus the general populations may be that these early-stage red cells have higher G6PD activity, leading to underestimation of G6PDd and over estimation of activity levels [34,35]. Importantly, no similar association was observed for *P. falciparum*. These findings, along with the absence of G6PDd in the Indigenous population, which also experienced the highest burden of *P. vivax* infection, provide the first epidemiological evidence of the protective association between G6PDd and *P. vivax* infection in an Afro-descendant population. The findings are consistent with the species-specific biology of G6PDd, in which *P. vivax* preferentially invades reticulocytes and other young red blood cells, potentially due to the higher GP6D activity in these cells [11,35].

Previous studies evaluating the association between G6PDd and malaria have yielded inconsistent results, probably reflecting differences in study populations, *Plasmodium* species, G6PD variants, phenotypic classification methods, and clinical presentation of infection [3]. Overall, the evidence supporting a protective effect of G6PDd has been stronger for *P. falciparum* [36–39] than for *P. vivax* [27,28,40–42]. Nevertheless, several studies have shown that severe G6PD variants, including the Mahidol [40,41], Viang [42], and Mediterranean [27,28] variants, are associated with lower *P. vivax* parasite density, a reduced risk of uncomplicated *P. vivax* malaria, or decreased disease severity in Southeast Asia [27,28,40–42]. In contrast, a study from Ethiopia found no association between G6PDd and *P. vivax* infection, although it was not powered to evaluate this relationship [43]. Considering the substantial geographic overlap between *P. vivax* transmission and G6PDd in the Americas and East Africa, further studies integrating quantitative phenotyping, genotyping, and longitudinal follow-up are needed to confirm this association and determine whether the magnitude of protection varies according to specific G6PD variants.

Despite the high prevalence of G6PDd in the Afro-Colombian population included in this study, self-reported events of primaquine-associated hemolysis were uncommon. Although history of hemolysis is subject to recall bias, it is notable given the extensive use of low-dose primaquine in the study area without prior G6PD testing. This finding is consistent with the experience reported in countries in Latin American and the Caribbean, where low-dose primaquine has long been administered without routine G6PD screening and clinically recognized hemolytic events have been infrequently reported [44,45]. It may also reflect to G6PD-deficient individuals experiencing fewer symptomatic *P. vivax* infections over time, resulting in lower cumulative exposure to low dose primaquine. Nonetheless, treatments with higher hemolytic potential should be used with caution with Afro-Colombian populations. For instance, any rollout of high-dose primaquine or tafenoquine in this group should be accompanied byG6PD testing and robust pharmacovigilance. In contrast, the Indigenous communities surveyed, which experienced the highest burden of *P. vivax* infection, may represent potential settings for the initial implementation of these radical cure strategies, provided that G6PD testing is performed according to WHO recommendations. More broadly, our findings illustrate that substantial heterogeneity in G6PDd prevalence may exist between population groups living within the same endemic area. Therefore, local epidemiology of both G6PDd and *P. vivax* transmission should be considered when planning the phased implementation of radical cure interventions, allowing countries to better balance treatment benefits against the risk of hemolytic adverse events.

This study has several limitations that should be considered when interpreting the findings. First, the prevalence estimates are generalizable only to populations at risk of malaria in Quibdó, and the findings for the Indigenous population are limited to the *Emberá dobida* communities included in this survey. Additional studies are needed to characterize G6PDd in other indigenous groups across Colombia. As the manufacturer-recommended thresholds for the STANDARD™ G6PD Test reduce false-negative classifications at the expense of increasing false positives compared with classifications based on the adjusted male median G6PD level [46], current estimates may overestimate prevalence. Use of the adjusted male median approach resulted in a larger proportion of females being classified as having intermediate G6PD activity. Second, women were overrepresented in the general population sample because many men were unavailable during household visits, which may have led to a slight underestimation of the overall prevalence of G6PDd given its higher frequency among males. Third, the study was not designed or powered to evaluate the association between G6PDd and *Plasmodium* infection, and causal inferences cannot be made. Finally, some of the data, such as the aforementioned history of hemolytic episodes, relied on self-report and were therefore subject to recall bias. However, health records may be unreliable in this resource-limited context, and the use of highly specific questions regarding acute hemolysis symptoms (e.g., dark urine and jaundice) provides valuable contextual information. It was also found that individuals with G6PDd were more likely to report history of malaria; we hypothesize that participants may have been more likely torecall clinical malaria episodes caused by *P. falciparum,* which was the predominant species among outpatients.

Despite these limitations, this study provides the first comprehensive characterization of G6PDd among Afro-Colombian and Indigenous populations living in a malaria-endemic region of Colombia. The findings reinforce the importance ofimplement G6PD testing in the Afro-Colombian population before the use of 8-aminoquinolines or other medications associated with a higher risk of hemolysis, in accordance with current WHO recommendations, while highlighting the substantial heterogeneity in G6PDd prevalence that may exist among population groups living within the same endemic area. This information can support countries in planning the phased implementation of radical cure strategies by identifying settings in which the balance between treatment benefits and the risk of hemolytic adverse events may differ according to the local epidemiology of both G6PDd and *P. vivax* [47]*.* Finally, future studies integrating quantitative phenotyping with molecular characterization of G6PD variants and prospective evaluation of treatment outcomes will be important to refining risk stratification [47] and optimizing radical cure strategies, including mass drug administration [48], in heterogeneous malaria-endemic settings.

## Supporting information

S1 Checklist Policy on inclusivity in global research. (PDF)

S1 Appendix Supplementary tables and figures. (DOCX)
