# Supplementary material for: Prevalence of glucose-6-phosphate dehydrogenase (G6PD) deficiency in a malaria endemic region of Colombia: Implications for radical cure of Plasmodium vivax
Source: PLOS Glob Public Health. 2026 Nov 11;6(11):e0007460. doi: 10.1371/journal.pgph.0007460 (PMC13754112; doi:10.1371/journal.pgph.0007460)
Supplement: Supplementary file 2 [file pgph.0007460.s002.docx]

**S1 Appendix. Supplementary tables and figures**

**Table A. Prevalence of *Plasmodium* spp. infection, by study group, ethnicity, sex and history of malaria.**

|  | **General population, Group 1** | **Indigenous population, Group 2** | **Outpatients,**  **Group 3** |
| --- | --- | --- | --- |
|  | n=1267 | n=194 | n=542 |
|  | % *Plasmodium* positive  (95% CI) | % *Plasmodium* positive (95% CI) | % *Plasmodium* positive (95% CI) |
| **Ethnicity (self-report)** |  |  |  |
| Afro-Colombian | 17.8% (13.5–23.0%) |  | 41.2% (36.3–46.2%) |
| Indigenous | 48.3% (5.3–94.0%) | 41.1% (34.2–48.5%) | 65.4% (55.3–74.3%) |
| Other | 11.2% (2.3–40.0%) |  | 67.6% (50.1–84.4%) |
| **Sex** | | | |
| Female | 10.7% (8.2–14.0%) | 38.5% (29.2–48.5%) | 42.8% (36.9–48.9%) |
| Male | 28.6% (19.8–39.0%) | 43.3% (33.0–54.1%) | 52.8% (46.6–58.8%) |
| **Residence and travel history** | | | |
| Residence in the study location at least one year | 18.1% (13.9–23.5%) | 40.8% (33.8–48.1%) | 46.4% (41.9–50.9%) |
| Slept at least one night outside the residence in a place with malaria transmission (in last 3 months) | 36.3% (11.7–71%) |  | 51.0 (43.6–58.5%) |
| **Clinical history of malaria** | | | |
| Previous diagnosis of malaria | 19.4% (14.6–25.5%) | 43.8% (34.8–53.1%) | 54.5% (49.5–59.4%) |
| Less than six months since prior episode of malaria | 11.3% (3.8–28.6%) | 52.3% (36.6–67.7%) | 72.9% (66.2–78.7%) |

* Estimates consider sample weights

**Table B. Self-reported responses to questions relating to prior history of hemolysis and adverse events (AE) after intake of primaquine (PQ), by G6PD measured in the study.**

|  | Deficient | Intermediate | Normal |
| --- | --- | --- | --- |
|  | (n=172) | (n=145) | (n=1675) |
| Prior diagnosis of G6PD deficiency | 0 (0%) | 0 (0%) | 0 (0%) |
| Prior diagnosis of hemolytic anemia | 1 (0.6%) | 2 (1.4%) | 19 (1.1%) |
| Prior hospitalization due to a hemolytic event^₤^ | 0 (0%) | 1 (50%) | 3 (15.8%) |
| Prior history of blood transfusion | 6 (3.5%) | 4 (2.8%) | 44 (2.6%) |
| PQ intake with last episode of malaria | 43 (25%) | 20 (13.8%) | 450 (26.9%) |
| Adverse event to PQ in last episode of malaria | 3 (7%) | 4 (20%) | 54 (12%) |
| Adverse event symptoms |  |  |  |
| Dizziness | 2 (66.7%) | 2 (50%) | 34 (63%) |
| Vomiting | 1 (33.3%) | 2 (50%) | 15 (27.8%) |
| Other* | 0 (0%) | 0 (0%) | 5 (9.3%) |

^₤^ Percentage calculated among participants who had prior diagnosis of hemolytic anemia

*Other included: malaise (n=1), nausea (n=1), diarrhea (n=1), pruritis (n=1), diarrhea with vomiting and cramps (n=1), all participants denied dark urine and jaundice.

**Table C. Prevalence of *Plasmodium* infection by G6PD status and study group.**

|  | **General population** | | | **Outpatients** | | | **Outpatients restricted to Afro-Colombians** | | |
| --- | --- | --- | --- | --- | --- | --- | --- | --- | --- |
|  | **Normal** | **Int** | **Deficient** | **Normal** | **Int** | **Deficient** | **Normal** | **Int** | **Deficient** |
|  | % (95% CI) | % (95% CI) | % (95% CI) | % (95% CI) | % (95% CI) | % (95% CI) | % (95% CI) | % (95% CI) | % (95% CI) |
| No malaria | 78.4% (72.2–83.5%) | 95.3% (86.2–98.5%) | 98.4% (95.9–99.4%) | 51.4% (46.8–55.9%) | 59.1% (36.7–78.5%) | 62.2% (44.8–77.1%) | 58.4% (52.9–63.7%) | 63.2% (38.6–82.8%) | 62.2% (44.8–77.1%) |
| *Plasmodium* spp. | 6.7% (4.7–9.4%) | 1.5% (0.4–6.0%) | 0.2% (0.0–1.8%) | 3.1% (1.8–5.3%) |  |  | 2.9% (1.5–5.5%) |  |  |
| *P. falciparum* | 1.2% (0.5–2.7%) | 0.0% (0.0–0.0%) | 1.4% (0.5–3.8%) | 26.0% (22.2–30.2%) | 27.3% (11.6–50.4%) | 32.4% (18.6–49.9%) | 26.5% (22.0–31.6%) | 26.3% (10.1–51.4%) | 32.4% (18.6–49.9%) |
| *P. vivax* | 13.7% (9.0–20.4%) | 3.1% (0.6–13.9%) | 0.0% (0.0–0.0%) | 19.5% (16.1–23.4%) | 13.6% (3.6–36.0%) | 5.4% (0.9–19.5%) | 12.1% (8.9–16.2%) | 10.5% (1.8–34.5%) | 5.4% (0.9–19.5%) |

**Table D. Associations between sex, ethnicity, malaria, and the level of G6PD enzymatic activity (IIU/g Hb), study population.**

|  | ***exp(β) adjusted* (general population, group 1, n=1263)** | |  | ***exp(β) adjusted* (Outpatients with suspected malaria, group 3, n=539)** | |  | ***exp(β) adjusted* (Outpatients with suspected malaria restricted to Afro-Colombians, n=397)** | |
| --- | --- | --- | --- | --- | --- | --- | --- | --- |
| *Factors* | *Estimates* | *CI 95%* |  | *Estimates* | *CI 95%* |  | *Estimates* | *CI 95%* |
| **Afro-Colombian** | 1.04 | 0.83 – 1.31 |  | 0.80 | 0.74 – 0.86 |  |  |  |
| **Male** | 0.91 | 0.85 – 0.97 |  | 0.90 | 0.85 – 0.97 |  | 0.89 | 0.82 – 0.97 |
| **Prior history of malaria** | 0.95 | 0.89 – 1.02 |  | 1.01 | 0.93 – 1.09 |  | 0.99 | 0.90 – 1.10 |
| **Current *Plasmodium* infection, any species** | 1.28 | 1.19 – 1.37 |  | 1.00 | 0.93 – 1.07 |  | 1.00 | 0.92 – 1.09 |
| *P. falciparum* | 1.17 | 0.95 – 1.44 |  | 0.98 | 0.90 – 1.06 |  | 0.95 | 0.86 – 1.05 |
| *P. vivax* | 1.29 | 1.19 – 1.40 |  | 1.12 | 1.03 – 1.23 |  | 1.10 | 0.96 – 1.26 |
| Others | 1.28 | 1.17 – 1.40 |  | 1.08 | 0.88 – 1.32 |  | 1.10 | 0.84 – 1.44 |

*Adjusted by ethnic group, sex and prior history of malaria.

exp(β): Should be interpreted as a percent of the increase (or decrease in other case if <1) of the mean or expected values of G6PD enzymatic

**Fig A. Comparing first and second measurements of G6PD enzymatic activity, Bland-Altman plots show 96.7% (238/246) agreement**

**Fig B. Comparing first and second measurements of hemoglobin, Bland-Altman plots show 97.8% (226/231).**

**Fig C. Boxplot with jittered data of G6PD activity in the among the three study populations**. Median and Interquartile Range (IQR) shown. Whiskers extend to 1.5* the IQR. General population (Group 1, red), indigenous communities (Group 2, blue), outpatients (Group 3, black) and outpatients restricted to Afro-Colombians (Group 3 sub-population, green).
