# Supplementary material for: Prevalence of glucose-6-phosphate dehydrogenase (G6PD) deficiency in a malaria endemic region of Colombia: Implications for radical cure of Plasmodium vivax
Source: PLOS Glob Public Health. 2026 Nov 11;6(11):e0007460. doi: 10.1371/journal.pgph.0007460 (PMC13754112; doi:10.1371/journal.pgph.0007460)
Supplement: Supplementary file 3 [file pgph.0007460.s004.docx]

12^th^ July 2026

Dr. Shazia Ruybal-Pesántez
Academic Editor
PLOS Global Public Health

Dear Editor,

Manuscript: "Prevalence of glucose-6-phosphate dehydrogenase (G6PD) deficiency in a malaria endemic region of Colombia: implications for radical cure of Plasmodium vivax" (PGPH-D-26-00377)

Thank you for the opportunity to revise and resubmit our manuscript. The reviewers gave positive and constructive feedback. We provide detailed responses to all of their comments below. We believe that the revised manuscript addresses all of the concerns and suggestions raised by the reviewers and it has improved significantly.

We hope that the manuscript is now acceptable for publication.

Yours sincerely,

**Lina M. Zuluaga-Idárraga**

**Professor of School of Public Health, University of Antioquia**

On behalf of all co-authors

**Response to reviewers**

**Editorial points:**

*There were some concerns raised with respect to the methodological details and the clarity of the discussion and interpretation of the manuscript findings. In particular, more details regarding G6PD methodology, including testing details, cutoffs, WHO classification, and interpretation of G6PD activity need to be clarified. All reviewers highlighted the need to improve the structure and readability of the Discussion section.*

*We welcome a revision that addresses the reviewer comments in full, particularly increasing clarity on the methodology, improvements to the Discussion section, and ensuring all data and associated code and scripts are publicly available to ensure reproducibility. We note that to be suitable for publication in PLOS Global Public Health your manuscript must be in line with the* [*PLOS Data policy*](https://track.editorialmanager.com/CL0/https:%2F%2Fjournals.plos.org%2Fglobalpublichealth%2Fs%2Fdata-availability/1/010f019e16d97ba7-61436c77-6f57-47b4-af54-03cf849923d9-000000/EC5ZZDGxPc5lgeR0Hgzc3YWZD1R7aPrvkiM20OXPZdc=258) *requiring authors "to make all data underlying the findings described in the manuscript fully available without restriction, with rare exceptions". Please do review our policy in full as you prepare your resubmission.*

**Response:** Thank you for this general comment. We have carefully revised the manuscript to address all the points raised by the reviewers. Specifically, we incorporated the suggested methodological details into the Abstract and Methods section. In addition, we thoroughly reviewed and changed the Discussion section to improve clarity and strengthen the interpretation of the findings.

To ensure reproducibility and compliance with the PLOS Global Public Health Data Policy, we have developed a data and code sharing plan consistent with current open science best practices. We deposit the fully anonymized study dataset together with a data dictionary in Zenodo (<https://zenodo.org/records/21404911>), an open-access repository operated by CERN and supported by the European Commission, which is explicitly listed among the interdisciplinary repositories recommended by PLOS. Zenodo provides a citable DOI for each deposit, supports CC BY licensing as required by the journal, and ensures long-term preservation of research outputs. Furthermore, all analytical code used to generate the study results, including the R scripts for tables and figures, are publicly available through a GitHub repository under an MIT open-source license and linked to Zenodo DOI <https://doi.org/10.5281/zenodo.21367641> . This dual-repository structure—Zenodo for data and GitHub/Zenodo for analytical code—aligns with current open science standards and fulfills the PLOS requirements for data availability, transparency, and reproducibility.

**Journal Requirements**

**1.	Please ensure that the Title in your manuscript file and the Title provided in your online submission form are the same.**

**Response:** Thank you for this observation. We have verified that the title in the manuscript file and the title provided in the online submission system are identical and fully consistent.

2.	**Please include a complete copy of PLOS’ questionnaire on inclusivity in global research in your revised manuscript. Our policy for research in this area aims to improve transparency in the reporting of research performed outside of researchers’ own country or community. The policy applies to researchers who have travelled to a different country to conduct research, research with Indigenous populations or their lands, and research on cultural artefacts. The questionnaire can also be requested at the journal’s discretion for any other submissions, even if these conditions are not met. Please find more information on the policy and a link to download a blank copy of the questionnaire here: https://journals.plos.org/globalpublichealth/s/best-practices-in-research-reporting. Please upload a completed version of your questionnaire as Supporting Information when you resubmit your manuscript.**

**Response:** Thank you for this guidance. We have completed the PLOS Questionnaire on Inclusivity in Global Research in full. In the revised manuscript, we included a dedicated section addressing these considerations and referenced the completed questionnaire as Supporting Information. The completed questionnaire will be uploaded to the submission platform along with the revised manuscript

**3.	Please provide separate figure files in .tif or .eps format. For more information about figure files please see our guidelines:**

**https://journals.plos.org/globalpublichealth/s/figures**

**https://journals.plos.org/globalpublichealth/s/figures#loc-file-requirements**

**Response:** Thank you for this comment. We have reviewed all figure files and adjusted their format according to the journal's requirements. Separate figure files have been prepared and will be submitted in the appropriate formats specified by the journal (.tif or .eps). We used NAAS tool to test all figure files.

**4**. **Please amend your detailed Financial Disclosure statement. This is published with the article. It must therefore be completed in full sentences and contain the exact wording you wish to be published.**

A. State the initials, alongside each funding source, of each author to receive each grant.

B. State what role the funders took in the study. If the funders had no role in your study, please state: “The funders had no role in study design, data collection and analysis, decision to publish, or preparation of the manuscript.”

**Response:** Thank you for this comment. The study was entirely supported by a single funding source, and no author received any additional specific funding related to this work. Therefore, we consider the original funding statement to be appropriate and consistent with the support received for the study. In addition, we have included the recommended statement specifying that: “The funders had no role in study design, data collection and analysis, decision to publish, or preparation of the manuscript” (lines 572-573).

**5.	"Some material included in your submission may be copyrighted. According to PLOS’s copyright policy, authors who use figures or other material (e.g., graphics, clipart, maps) from another author or copyright holder must demonstrate or obtain permission to publish this material under the Creative Commons Attribution 4.0 International (CC BY 4.0) License used by PLOS journals. Please closely review the details of PLOS’s copyright requirements here: PLOS Licenses and Copyright. If you need to request permissions from a copyright holder, you may use PLOS's Copyright Content Permission form.**

**Please respond directly to this email or email the journal office and provide any known details concerning your material's license terms and permissions required for reuse, even if you have not yet obtained copyright permissions or are unsure of your material's copyright compatibility.**

**Potential Copyright Issues:**

**Figure 1: please (a) provide a direct link to the base layer of the map (i.e., the country or region border shape) and ensure this is also included in the figure legend; and (b) provide a link to the terms of use / license information for the base layer image or shapefile. We cannot publish proprietary or copyrighted maps (e.g. Google Maps, Mapquest) and the terms of use for your map base layer must be compatible with our CC-BY 4.0 license.**

**Note: if you created the map in a software program like R or ArcGIS, please locate and indicate the source of the basemap shapefile onto which data has been plotted.**

**If your map was obtained from a copyrighted source please amend the figure so that the base map used is from an openly available source. Alternatively, please provide explicit written permission from the copyright holder granting you the right to publish the material under our CC-BY 4.0 license.**

**Please note that the following CC BY licenses are compatible with PLOS license: CC BY 4.0, CC BY 2.0 and CC BY 3.0, meanwhile such licenses as CC BY-ND 3.0 and others are not compatible due to additional restrictions.**

**If you are unsure whether you can use a map or not, please do reach out and we will be able to help you. The following websites are good examples of where you can source open access or public domain maps:**

*** U.S. Geological Survey (USGS) - All maps are in the public domain. (http://www.usgs.gov)**

*** PlaniGlobe - All maps are published under a Creative Commons license so please cite “PlaniGlobe, http://www.planiglobe.com, CC BY 2.0” in the image credit after the caption. (http://www.planiglobe.com/?lang=enl)**

*** Natural Earth - All maps are public domain. (http://www.naturalearthdata.com/about/terms-of-use/)**

**If the reviewer comments include a recommendation to cite specific previously published works, please review and evaluate these publications to determine whether they are relevant and should be cited. There is no requirement to cite these works unless the editor has indicated otherwise.**

**Please review your reference list to ensure that it is complete and correct. If you have cited papers that have been retracted, please include the rationale for doing so in the manuscript text, or remove these references and replace them with relevant current references. Any changes to the reference list should be mentioned in the rebuttal letter that accompanies your revised manuscript. If you need to cite a retracted article, indicate the article’s retracted status in the References list and also include a citation and full reference for the retraction notice.**

**Response:** Thank you for your detailed comments regarding the copyright requirements for Figure 1.

Figures 1A and 1B were created using the open-source software QGIS version 3.28. The basemap shapefiles were obtained from official Colombian government sources.

Specifically, Figure 1A was created using the municipal boundaries shapefile provided by the Colombian National Administrative Department of Statistics (DANE), available at https://geoportal.dane.gov.co/servicios/descarga-y-metadatos/datos-geoestadisticos/. The DANE Geoportal provides geospatial data under a Creative Commons license (CCBY4.0), and the corresponding license and terms of use are available at https://geoportal.dane.gov.co/acerca-del-geoportal/licencia-y-condiciones-de-uso/#gsc.tab=0.

Figure 1B was created using a map available on the official website of the Municipality of Quibdó, available at https://www.quibdo-choco.gov.co/MiMunicipio/Paginas/Informacion-del-Municipio.aspx. We were unable to identify specific licensing or terms of use for this cartographic material on the municipality's official website.

For both figures, the original maps were adapted to comply with the journal's formatting requirements. We have revised the figure legends to include the corresponding cartographic source, the direct link to the original basemap, and, where available, the applicable licensing information (lines 127 – 133).

The maps are intended solely for illustrative purposes. Figure 1A depicts the location of the study area, whereas Figure 1B illustrates the proportional distribution of the study sample within the municipality. No spatial analyses were performed using these maps, and no geospatial inferences are presented in the manuscript. Therefore, the figures do not include geographic coordinates, datum, projection information, or georeferenced files, as these elements are not required for their intended illustrative purpose.

We hope that the additional information provided in the revised figure legends and in this response adequately addresses the journal's copyright and attribution requirements.

**Reviewer #1:**

**Overall comment**

This is a well-conducted and important study addressing a key evidence gap on G6PD deficiency in a malaria-endemic setting in Latin America. However, the manuscript would benefit from improvements in the Discussion section, which is currently difficult to follow.

**Response:** We sincerely thank the reviewer for this positive assessment of our study and for recognizing its contribution to addressing an important evidence gap on G6PD deficiency in a malaria-endemic setting in Latin America.

In response to this valuable suggestion, we have thoroughly revised and reorganized the Discussion section to improve its clarity, coherence, and overall readability. Specifically, we have strengthened the interpretation of the main findings, improved the logical flow between paragraphs, reduced redundancy, and clarified the clinical and public health implications of our results in the context of the existing literature. We believe that these revisions have substantially enhanced the readability and scientific discussion of the manuscript.

**Major Comments**

**1. Abstract: clarify G6PD methodology**

**The abstract would benefit from briefly specifying:**

**• which G6PD test was used, and**

**• how deficient, intermediate, and normal activity were defined**

**Response:** We thank the reviewer for this helpful suggestion. In response, we have revised the Abstract to provide greater methodological clarity. Specifically, we have included the name of the quantitative G6PD test used in the study and indicated activity threshold used to classify G6PD activity.

Lines 42 – 44 now read: *“Levels of G6PD activity (IU/g hemoglobin) were classified as deficient (≤ 4.0), intermediate (4.1–6.0 in females), or normal (≥ 6.1 in females, ≥ 4.1 in males), and the prevalence of G6PDd was calculated.”*

**2. Terminology: “standard primaquine” vs “low-dose primaquine”**

**In the Introduction, the manuscript refers to “standard-dose primaquine.” Given that WHO guidelines now recommend high-dose primaquine in many settings, the term “standard” is ambiguous and potentially outdated. It would be clearer and more consistent to refer to this regimen throughout as “low-dose primaquine.”**

**Response:** We thank the reviewer for this important observation and fully agree with the recommendation. Referring to the regimen as "standard-dose primaquine" was an oversight on our part and may indeed be ambiguous given current WHO recommendations, which include higher-dose primaquine regimens in many settings.

Accordingly, we have revised the manuscript throughout to replace the term "standard-dose primaquine" with "low-dose primaquine" (line 86), ensuring consistency with current terminology and WHO guidance.

**3. Methods: G6PD testing**

**The section describing G6PD testing raises several points that require clarification:**

**• The manuscript describes repeat testing and notes that participants with Hb <7 g/dL should not have G6PD results interpreted.**

**• However, it is not clear:**

**o how repeat test results were handled in the analysis**

**o whether participants with Hb <7 g/dL were excluded, and if so, from which analyses**

**This information should be clearly stated in the Methods, rather than only becoming apparent later in the Results.**

**Response:** Thank you for this valuable comment. We fully agree that these methodological details should have been clearly described in the Methods section. In response, we have revised the G6PD activity testing subsection to explicitly describe how repeated G6PD measurements were handled in the analyses and to clarify the treatment of participants with hemoglobin (Hb) concentrations <7 g/dL (line 217 and lines 220 – 224).

According to the manufacturer's instructions for the STANDARD™ G6PD Test, although the analyzer may generate a G6PD result when the Hb concentration is <7 g/dL, these results should not be interpreted. Therefore, participants with Hb concentrations <7 g/dL should be excluded from analyses involving G6PD activity or G6PD status. We acknowledge that we inadvertently retained these observations in our original analyses, which was an error on our part.

To address the reviewer's comment and correct this error, we repeated all relevant analyses after excluding participants with Hb concentrations <7 g/dL. The revised manuscript now includes the updated results in Tables 2 and 3, Figures 3, 4, and 5, and Supporting Information Tables S3, S4, and S5. The corresponding methodological description has also been added to the Methods section to ensure that the analytical approach is clearly explained (lines 220 – 224).

**4. Reliability of self-reported adverse events**

**I am a bit doubtful if this section adds much and would be inclined to remove from the manuscript. Self-reported events are notoriously difficult to interpret for recall bias etc. At a minimum it would be good to add over which timeframe these events were recalled. It’s not clear how this data supports any of the main messages in the manuscript.**

**Response:** Thank you for this thoughtful comment. We appreciate the reviewer's concern regarding the potential for recall bias in self-reported adverse events, and we fully acknowledge this as an important limitation of these data. Nevertheless, after careful consideration, we believe that this section provides valuable contextual information and have therefore decided to retain it in the manuscript.

To improve clarity, we have revised the Methods section to specify that participants were asked about adverse events from last malaria episode (majority it was during last 6 months) (lines 202 -203). We also emphasize in the Discussion that these findings should be interpreted with caution because they are based on self-reported information and are therefore subject to recall bias (lines 534 – 538).

Importantly, the adverse events included in our questionnaire were not selected arbitrarily. Based on the published literature, we identified the most common adverse events associated with 8-aminoquinolines and developed a standardized checklist of signs and symptoms. Field staff received specific training on how to administer this questionnaire, with particular emphasis on symptoms suggestive of acute hemolysis, including dark urine and jaundice.

Although we recognize the possibility of imperfect recall, malaria is a well-recognized illness in this endemic population, and the key manifestations of acute hemolysis are distinctive clinical events that participants are generally able to recall when appropriate, standardized questioning is used. We believe these data therefore provide useful, albeit indirect, evidence.

The relevance of these findings is that, in a population with a high prevalence of G6PD deficiency where low-dose primaquine has historically been administered without prior G6PD testing, self-reported hemolytic adverse events were uncommon. This observation is also consistent with the experience reported by local healthcare providers. While these findings should not be interpreted as definitive evidence of drug safety, they provide supportive real-world evidence that the risk of clinically recognized hemolytic events associated with low-dose primaquine appears to be low in this setting. Furthermore, they are consistent with one of the manuscript's principal findings—that G6PD deficiency may be associated with a lower risk of *P. vivax* infection—suggesting that individuals with G6PD deficiency in this population have historically low exposition to primaquine since they could not have symptomatic infections.

**5. Discussion: structure and readability**

**The Discussion is currently difficult to follow it feels disjointed from the rest of the manuscript. It would benefit from tightening overall to avoid repetition.**

**The first paragraph largely repeats numerical results, rather than providing a clear interpretative summary.**

**It is a bit confusing to jump between epidemiology / prevalence comparisons in the second paragraph to test inherent issues in the third paragraph back to prevalence among indigenous populations in the 4th paragraph. Perhaps it would make it easier on the reader to talk about prevalence findings first, then about measurement and classification issues.**

**Similarly the key discussion section on relationship of G6PD and malaria are difficult to read (paragraph 5, 6 7 and 8) and could be tighter.**

**Response:** Thank you for this thoughtful and constructive comment. We agree that the original Discussion could be improved to provide a clearer and more coherent interpretation of our findings. We believe these revisions have resulted in a more focused, logical, and cohesive Discussion that is better aligned with the overall structure of the manuscript.

**6. Interpretation of policy implications (currently paragraph 10 of the discussion and conclusions)**

**In several places, the manuscript refers to implications for policy, but these are often vague or rather supportive of current guidance than new.**

**For example:**

**• 532 and 571: The suggestion that findings support the need for G6PD testing prior to high-dose primaquine or tafenoquine is not novel—this is already established WHO guidance. The presented data may support or reinforce this approach, but do not introduce a new policy direction.**

**Towards the end of the Discussion, the manuscript appears to suggest that approaches to radical cure and MDA might differ for the indigenous population. Are the authors suggesting that in these populations high dose primaquine or tafenoquine could be used without testing?**

**Response:** Thank you for this insightful comment. We agree with the reviewer that our findings do not introduce a new policy direction but rather provide additional evidence supporting the current WHO recommendations regarding G6PD testing before the administration of high-dose primaquine or tafenoquine.

At the time this study was conducted, Colombia had not yet initiated the implementation of the updated WHO recommendations for the radical cure of P. vivax, including high-dose primaquine and tafenoquine. Therefore, we believe that our findings are relevant because they help identify the epidemiological settings in which these treatment strategies will need to be introduced. Given the marked heterogeneity of malaria transmission and population composition across Colombia, our estimates of G6PD deficiency prevalence provide important information for planning the phased implementation of these new treatment options and anticipating the operational requirements for G6PD testing in different regions.

We also appreciate the opportunity to clarify the final message of the Discussion. Our intention was not to suggest that high-dose primaquine or tafenoquine could be used without prior G6PD testing in Indigenous populations. We fully support the WHO recommendation that G6PD testing should precede the use of these regimens whenever they are prescribed. Rather, we intended to convey that populations with a very low prevalence of G6PD deficiency may represent more favorable settings for the initial implementation of these treatment strategies because, when G6PD testing is performed as recommended, the expected risk of hemolytic adverse events is lower.

Conversely, our findings also highlight the importance of determining the prevalence of G6PD deficiency in populations with greater genetic admixture, where the frequency of G6PD deficiency may be substantially higher. Such information is essential for accurately assessing the balance between the benefits and risks of radical cure strategies and for informing implementation planning in different epidemiological contexts. To avoid misinterpretation, we have revised the Discussion to clarify these points and to ensure that our conclusions are fully aligned with current WHO guidance.

**Minor comments:**

**Introduction**

**• Line 99: missing closing bracket**

**Response:** corrected

**• Line 110: missing space**

**Response:** corrected

**Methods**

**• Line 137: add “febrile” for consistency with terminology used elsewhere**

**Response:** corrected

**Results**

**• Line 354: “primaquine” is abbreviated as “PQ” without prior definition; ensure consistency throughout**

**Response:** corrected; the abbreviation PQ was removed from the entire manuscript.

**• In line 474, the manuscript refers to a “dose-dependent effect” in relation to G6PD activity. This terminology is not appropriate in this context, as G6PD activity is a biological measure, not an administered exposure**

**Response:** it was deleted from discussion section.

**Reviewer #2:**

1. **The manuscript is well written and provides a useful piece of evidence on prevalence of G6PD deficiency in different populations of Colombia. I only have minor comments and suggestions to improve the manuscript.**

**Throughout the text it is mentioned few times that G6PD activity might increase during a malaria episode; while this is expected and is partially explained by parasite-induced haemolysis, in P. vivax bone marrow suppression might counterbalance this phenomenon. At the moment, only few data have been collected properly on this issue so the statements in the text should be revised.**

**Response:** Thank you for your positive assessment of our manuscript and for recognizing its contribution to the understanding of G6PD deficiency prevalence in different populations in Colombia. We also appreciate this thoughtful comment regarding the interpretation of G6PD activity during acute *P. vivax* malaria.

We agree that the available evidence on changes in G6PD activity during malaria episodes remains limited and that the underlying mechanisms are not yet fully understood. We have revised the relevant paragraph in the Discussion to adopt a more cautious interpretation of the available evidence, acknowledging the uncertainty surrounding this phenomenon and avoiding statements that could overstate the current level of evidence.

2. **Table 2: prevalence of G6PDd in the indigenous group should not be reported as NA but as 0/N (where N is the total number of indigenous participants and females and males)**

**Response:** Thank you for this helpful suggestion. We appreciate the reviewer's recommendation to avoid reporting these values as "NA." After careful consideration, we decided to maintain a consistent format throughout the table. Instead of displaying "NA," we have left blank the cells where the corresponding estimate is not applicable. In addition, we have included a footnote specifying that estimates were not calculated for cells in which no participants with G6PD deficiency were identified. We believe this approach improves the readability of the table while maintaining consistency across all reported estimates.

3. **Table 2: the WHO classification is not meant to be used as a classification of individual activities; therefore, only the categorization in % of activity should be used without mention of WHO classification. The citation (26) used is not appropriate and should be removed. For more info on the recent WHO classification and its use on classification of G6PD variants, please see this link New WHO classification of genetic variants causing G6PD deficiency – PMC**

**Response:** Thank you for this helpful comment and for pointing out the distinction between the WHO classification of G6PD genetic variants and the classification of G6PD activity. We agree that referring to the activity categories as the "WHO classification" could be misleading. In response to your suggestion, we now refer to these categories as the "% activity classification."

Regarding Reference 26, we respectfully believe that it remains appropriate and should be retained. This reference is cited to support the calculation of the adjusted male median used to define G6PD activity thresholds, rather than the classification of G6PD genetic variants. For this reason, we do not believe that the recent WHO publication by Luzzatto et al. (2024) is the appropriate reference in this context, as our manuscript does not address the classification of G6PD genetic variants but rather the classification of enzyme activity based on the adjusted male median. We have therefore retained Reference 26 for this specific methodological purpose.

4. **Line 384 Can the authors explain why they choose to use normal AND intermediate as reference group?**

**When analysing G6PD activity in malaria patients and healthy population, I think it would make more sense to stick to the comparison of prevalence of different G6PD phenotypes in malaria cases vs control (healthy subjects). Using changes in G6PD activity levels might not be appropriate because the Biosensor is not a fully quantitative test, the level of G6PD activity and Hb are influenced by duration of infection and parasitemia which were factors not analysed.**

**Response:** Thank you for this important question and for highlighting this critical methodological aspect. We appreciate the opportunity to clarify both our analytical approach and the rationale for the definition of the outcome categories.

For the analyses exploring the association between G6PD deficiency prevalence and *P. vivax* infection, the outcome was defined as G6PD status, grouped into two categories: deficient versus normal/intermediate. Accordingly, the estimates presented in Table 3 correspond to prevalence ratios (PRs) for G6PD deficiency according to each explanatory variable, with the reference category for the exposure of interest being participants without *P. vivax* infection.

We acknowledge that, because G6PD deficiency is a genetically determined condition, an alternative and perhaps more intuitive analytical framework would be to consider *P. vivax* infection as the outcome and G6PD deficiency as the exposure. However, given that the relationship between these two variables may also be bidirectional, we believe that our approach is also valid. Moreover, it is consistent with the primary objective of the study, which was to estimate the prevalence of G6PD deficiency and to explore association with malaria.

As noted in the manuscript, these analyses address a secondary, exploratory objective and should therefore be interpreted with caution. In response to the reviewer's comment, we have strengthened the Discussion to emphasize the exploratory nature of these findings and to acknowledge that several factors not measured in this study—including parasite density, duration of infection, and other genetic and clinical characteristics—could act as confounders or effect modifiers of the observed association.

Regarding the decision to combine participants with intermediate and normal G6PD activity into the reference category, this choice was based on two main considerations.

First, from a biological perspective, if G6PD deficiency confers protection against *P. vivax* infection, such protection would be expected to occur primarily among individuals with markedly reduced enzyme activity rather than among those with intermediate activity, whose residual enzyme activity may still be sufficient to support normal parasite development within erythrocytes. Although the available evidence is limited, studies reporting a protective association (References 27, 28, and 43) have mainly involved genetic variants such as Mahidol and Mediterranean, which are characterized by substantial reductions in enzyme activity (typically <20% according to Pfeffer et al., 2022, https://doi.org/10.3390/pathogens11091045; and Nannelli et al., 2023, https://doi.org/10.1111/bjh.18943). Therefore, if a protective effect exists, it would be expected to be greatest among individuals with the lowest levels of G6PD activity.

Second, our G6PD phenotype classification followed the manufacturer's recommended thresholds for the STANDARD™ G6PD Test, which were developed to maximize patient safety when guiding radical cure treatment decisions. These thresholds are intentionally conservative and may classify some individuals with near-normal enzyme activity as intermediate or even deficient. Consequently, combining intermediate and deficient participants into a single category could increase outcome misclassification and bias the estimated associations toward the null. We therefore considered the comparison of deficient versus normal/intermediate participants to be the most biologically and methodologically appropriate approach for this exploratory analysis.

With respect to the reviewer's comment on the analysis of continuous G6PD activity, we agree that these results should be interpreted cautiously because G6PD activity measured with the biosensor may be influenced by factors related to acute malaria, including the duration of infection and parasite density, neither of which was assessed in this study. For this reason, we have revised the Discussion to place greater emphasis on the categorical analyses, while presenting the continuous analyses as supportive exploratory findings rather than primary evidence.

5. **The discussion is very long and could really be improved by removing information already given in the introduction and by streamlining the text by focusing on the main findings.**

**Response:** Thank you for this valuable suggestion. We agree that the Discussion was longer than necessary and contained some information already presented in the Introduction. In response, we have substantially revised this section by removing repetitive background information, reducing redundancy, and streamlining the text to focus more clearly on the main findings and their interpretation. We believe these revisions have improved the clarity, readability, and overall flow of the Discussion.

**Reviewer #3:**

**Thanks to the Editor and authors for the opportunity to review this interesting manuscript. Zuluaga-Idarraga et al investigate the prevalence of G6PD deficiency in the majority Afro-Columbian and minority Emberá dobida indigenous population in Quibdó, Colombia, as well as the association between G6PD activity and Plasmodium spp infection by PCR. G6PD deficiency was observed to be common among the predominantly Afro Colombian general population but absent in the indigenous group, which had the highest P. vivax burden.**

**Overall, the manuscript is clearly written, surveys and testing approaches are methodologically appropriate and the study provides relevant insights for guiding the targeted implementation of P. vivax radical cure. The analyses are appropriate and the findings are clearly presented, thoughtfully discussed and limitations noted.**

**Several minor revisions are suggested to improve clarity and interpretation of the study and it’s findings. I recommend acceptance pending the authors addressing these minor revisions.**

1. **The details of the quantitative G6PD test used should be provided in the abstract.**

**Response:** Thanks for the suggestion, we have included details of the test in the abstract.

2. **The cutoffs used to classify individuals as deficient, intermediate and normal should be provided in the abstract.**

**Response:** Thanks for the suggestion. As the Abstract is subject to word limitations, the specific activity thresholds used to define each category are presented in detail in the Methods section of the manuscript.

3. **Line 42-42 of abstract, should indicate that “The relationships between G6PD status, ethnicity and the risk of Plasmodium infection was evaluated.”**

**Response:** Thank you for this helpful suggestion. We appreciate the reviewer's perspective; however, we respectfully believe that the proposed wording does not accurately reflect the objective of our analysis.

As a secondary objective, this study explored the association between G6PD deficiency and malaria (both previous malaria history and current Plasmodium infection). Ethnicity and sex were included in the analyses because they are well-established factors associated with G6PD deficiency and were therefore considered as covariates for adjustment of the estimated associations, rather than exposures of primary interest. For this reason, we believe that stating that the study evaluated the relationships between G6PD status, ethnicity, and the risk of Plasmodium infection could be misleading, as it may imply that ethnicity was one of the primary variables under investigation.

We have therefore retained the original wording, which we believe more accurately reflects the study objectives and analytical approach.

4. **Line 74 – please clarify that G6PDd is important in determining the safety of radical cure for P. vivax rather for radical cure itself.**

**Response:** Thank you for the suggestion, we have included the change.

5. **Table 1 provides data on consanguinity between parents, however there are no methodological details provided as to how genetic relatedness was determined.**

**Response:** Thank you for this helpful comment. We agree that the method used to determine parental consanguinity should be clearly described.

In response, we have clarified this information in Table 1 by adding a footnote indicating that **parental consanguinity was determined based on participants' self-reported parental relatedness.** We believe this clarification improves the transparency of the methods used to collect this variable.

6. **The authors need to clarify how they are making the full dataset available as per journal requirements. The data availability statement indicates that all data are provided within the manuscript or supplementary information files, however this only includes summary statistics.**

**Response:** Thank you for raising this important point. We have addressed this issue in greater detail in our response to the Editor.

In accordance with the journal's data availability requirements, we have revised the Data Availability Statement. The complete de-identified dataset and all analytical code used to generate the study results, including the R scripts used to produce the tables and figures, will be made publicly available through a GitHub repository under an MIT open-source license. The repository also is archived in Zenodo to ensure long-term accessibility and reproducibility (<https://zenodo.org/records/21404911>). We believe these revisions fully comply with the journal's data sharing policy.
