# Supplementary material for: Prevalence of glucose-6-phosphate dehydrogenase (G6PD) deficiency in a malaria endemic region of Colombia: Implications for radical cure of Plasmodium vivax
Source: PLOS Glob Public Health. 2026 Nov 11;6(11):e0007460. doi: 10.1371/journal.pgph.0007460 (PMC13754112; doi:10.1371/journal.pgph.0007460)
Supplement: Supplementary file 4 [file pgph.0007460.s005.DOC]

9^th^ September 2026

Dr. Shazia Ruybal-Pesántez
Academic Editor
PLOS Global Public Health

Dear Editor,

Manuscript: "Prevalence of glucose-6-phosphate dehydrogenase (G6PD) deficiency in a malaria endemic region of Colombia: implications for radical cure of Plasmodium vivax" (PGPH-D-26-00377)

Dear Editors,

Thank you very much for your careful review of our manuscript and for the opportunity to submit a revised version.

In response to the comments received, we have carefully reviewed the entire manuscript and corrected grammatical and typographical errors throughout the document, rather than limiting our revisions to the specific errors identified during the review process. For clarity, we have also provided a point-by-point response detailing the revisions and corrections made in the manuscript in response to the comments received.

We hope that the manuscript is now acceptable for publication.

Yours sincerely,

**Lina M. Zuluaga-Idárraga**

**Professor of School of Public Health, University of Antioquia**

On behalf of all co-authors

**Response to reviewers**

**Journal Requirements:** No comments received.

**Additional Editor Comments:**

**The reviewers comments were addressed, however there are some grammatical and typographical errors that remain in this revised version. Please thoroughly check for any typos or grammatical errors throughout the manuscript, some examples below (these are non-exhaustive, please revise carefully):**

**Response:** we have carefully reviewed the entire manuscript and corrected grammatical and typographical errors throughout the document

**L37: Plasmodium should be fully written upon first mention and then abbreviated**

**Response:** Plasmodium vivax is fully written upon first mention on L33 and then is presented abbreviated (L34, L37).

**L52: "respectively" repeated**

**Response:** eliminated

L149: outpatients febrile -> febrile outpatients

**Response:** corrected

L442: Value were not estimated because some categories were not contained G6PDd cases -> Values were not estimated because some categories did not contain G6PDd cases.

**Response:** corrected.

L481: statistically difference -> statistically different

**Response:** corrected.

L493: vivax -> P. vivax

**Response:** corrected.

**Additional changes:** A thorough review of the entire manuscript was conducted to correct grammatical and spelling errors, and all changes have been highlighted in the document**.**

**Reviewers' comments:** No comments received.

**Figure Resubmissions:**

**While revising your submission, we strongly recommend that you use PLOS’s NAAS tool (https://ngplosjournals.pagemajik.ai/artanalysis) to test your figure files. NAAS can convert your figure files to the TIFF file type and meet basic requirements (such as print size, resolution), or provide you with a report on issues that do not meet our requirements and that NAAS cannot fix.**

**After uploading your figures to PLOS’s NAAS tool - https://ngplosjournals.pagemajik.ai/artanalysis, NAAS will process the files provided and display the results in the "Uploaded Files" section of the page as the processing is complete. If the uploaded figures meet our requirements (or NAAS is able to fix the files to meet our requirements), the figure will be marked as "fixed" above. If NAAS is unable to fix the files, a red "failed" label will appear above. When NAAS has confirmed that the figure files meet our requirements, please download the file via the download option, and include these NAAS processed figure files when submitting your revised manuscript.**

**Response:** All figures were validated using PLOS’s NAAS tool following our previous submission. We have confirmed that the figures comply with the journal’s specifications and requirements.
