# Supplementary material for: Prevalence of glucose-6-phosphate dehydrogenase (G6PD) deficiency in a malaria endemic region of Colombia: Implications for radical cure of Plasmodium vivax
Source: PLOS Glob Public Health. 2026 Nov 11;6(11):e0007460. doi: 10.1371/journal.pgph.0007460 (PMC13754112; doi:10.1371/journal.pgph.0007460)
Supplement: Peer review history

# Author comment: Author response to Decision Letter 0

**DOI:** 10.1371/journal.pgph.0007460.r001

**Transfer Alert**

This paper was transferred from another journal. As a result, its full editorial history (including decision letters, peer reviews and author responses) may not be present.

19 Feb 2026

# Peer review: Decision Letter 0

**DOI:** 10.1371/journal.pgph.0007460.r002

**Authors:** Shazia Ruybal-Pesántez

11 May 2026

PGPH-D-26-00377

Prevalence of glucose-6-phosphate dehydrogenase (G6PD) deficiencyin a malaria endemic malaria region of Colombia: implications for radical cure of Plasmodium vivax

PLOS Global Public Health

Dear Dr. Zuluaga-Idarraga,

Thank you for submitting your manuscript to PLOS Global Public Health. After careful consideration, we feel that it has merit but does not fully meet PLOS Global Public Health’s publication criteria as it currently stands. Therefore, we invite you to submit a revised version of the manuscript that addresses the points raised during the review process.

The reviewers found your work important in filling key gaps in the evidence base for G6PD deficiency in Latin America, with relevant implications for guiding *P. vivax*  radical cure implementation strategies. However there were some concerns raised with respect to the methodological details and the clarity of the discussion and interpretation of the manuscript findings. In particular, more details regarding G6PD methodology, including testing details, cutoffs, WHO classification, and interpretation of G6PD activity need to be clarified. All reviewers highlighted the need to improve the structure and readability of the Discussion section.

We welcome a revision that addresses the reviewer comments in full, particularly increasing clarity on the methodology, improvements to the Discussion section, and ensuring all data and associated code and scripts are publicly available to ensure reproducibility. We note that to be suitable for publication in PLOS Global Public Health your manuscript must be in line with the PLOS Data policy requiring authors "to make all data underlying the findings described in the manuscript fully available without restriction, with rare exceptions". Please do review our policy in full as you prepare your resubmission.

Please submit your revised manuscript by Jun 10 2026 11:59PM. If you will need more time than this to complete your revisions, please reply to this message or contact the journal office at globalpubhealth@plos.org. When you're ready to submit your revision, log on to https://www.editorialmanager.com/pgph/ and select the 'Submissions Needing Revision' folder to locate your manuscript file.

Please include the following items when submitting your revised manuscript:

- A letter that responds to each point raised by the editor and reviewer(s). You should upload this letter as a separate file labeled 'Response to Reviewers'.
- A marked-up copy of your manuscript that highlights changes made to the original version. You should upload this as a separate file labeled 'Revised Manuscript with Track Changes'.
- An unmarked version of your revised paper without tracked changes. You should upload this as a separate file labeled 'Manuscript'.

Guidelines for resubmitting your figure files are available below the reviewer comments at the end of this letter.

As the corresponding author, your ORCID iD is verified in the submission system and will appear in the published article. PLOS supports the use of ORCID, and we encourage all coauthors to register for an ORCID iD and use it as well. Please encourage your coauthors to verify their ORCID iD within the submission system before final acceptance, as unverified ORCID iDs will not appear in the published article. *Only*  the individual author can complete the verification step; PLOS staff *cannot*  verify ORCID iDs on behalf of authors.

We look forward to receiving your revised manuscript.

Kind regards,

Shazia Ruybal-Pesántez

Academic Editor

PLOS Global Public Health

Journal Requirements:

1. Please ensure that the Title in your manuscript file and the Title provided in your online submission form are the same.

1. Please include a complete copy of PLOS’ questionnaire on inclusivity in global research in your revised manuscript. Our policy for research in this area aims to improve transparency in the reporting of research performed outside of researchers’ own country or community. The policy applies to researchers who have travelled to a different country to conduct research, research with Indigenous populations or their lands, and research on cultural artefacts. The questionnaire can also be requested at the journal’s discretion for any other submissions, even if these conditions are not met. Please find more information on the policy and a link to download a blank copy of the questionnaire here: https://journals.plos.org/globalpublichealth/s/best-practices-in-research-reporting. Please upload a completed version of your questionnaire as Supporting Information when you resubmit your manuscript.

1. Please provide separate figure files in .tif or .eps format.
  For more information about figure files please see our guidelines:
  https://journals.plos.org/globalpublichealth/s/figures
  https://journals.plos.org/globalpublichealth/s/figures#loc-file-requirements

1. Please amend your detailed Financial Disclosure statement. This is published with the article. It must therefore be completed in full sentences and contain the exact wording you wish to be published.
  A. State the initials, alongside each funding source, of each author to receive each grant.
  B. State what role the funders took in the study. If the funders had no role in your study, please state: “The funders had no role in study design, data collection and analysis, decision to publish, or preparation of the manuscript.”

1. "Some material included in your submission may be copyrighted. According to PLOS’s copyright policy, authors who use figures or other material (e.g., graphics, clipart, maps) from another author or copyright holder must demonstrate or obtain permission to publish this material under the Creative Commons Attribution 4.0 International (CC BY 4.0) License used by PLOS journals. Please closely review the details of PLOS’s copyright requirements here: PLOS Licenses and Copyright. If you need to request permissions from a copyright holder, you may use PLOS's Copyright Content Permission form.

Please respond directly to this email or email the journal office and provide any known details concerning your material's license terms and permissions required for reuse, even if you have not yet obtained copyright permissions or are unsure of your material's copyright compatibility.

Potential Copyright Issues:

Figure 1: please (a) provide a direct link to the base layer of the map (i.e., the country or region border shape) and ensure this is also included in the figure legend; and (b) provide a link to the terms of use / license information for the base layer image or shapefile. We cannot publish proprietary or copyrighted maps (e.g. Google Maps, Mapquest) and the terms of use for your map base layer must be compatible with our CC-BY 4.0 license.

Note: if you created the map in a software program like R or ArcGIS, please locate and indicate the source of the basemap shapefile onto which data has been plotted.

If your map was obtained from a copyrighted source please amend the figure so that the base map used is from an openly available source. Alternatively, please provide explicit written permission from the copyright holder granting you the right to publish the material under our CC-BY 4.0 license.

Please note that the following CC BY licenses are compatible with PLOS license: CC BY 4.0, CC BY 2.0 and CC BY 3.0, meanwhile such licenses as CC BY-ND 3.0 and others are not compatible due to additional restrictions.

If you are unsure whether you can use a map or not, please do reach out and we will be able to help you. The following websites are good examples of where you can source open access or public domain maps:

* U.S. Geological Survey (USGS) - All maps are in the public domain. (http://www.usgs.gov)

* PlaniGlobe - All maps are published under a Creative Commons license so please cite “PlaniGlobe, http://www.planiglobe.com, CC BY 2.0” in the image credit after the caption. (http://www.planiglobe.com/?lang=enl)

* Natural Earth - All maps are public domain. (http://www.naturalearthdata.com/about/terms-of-use/)

If the reviewer comments include a recommendation to cite specific previously published works, please review and evaluate these publications to determine whether they are relevant and should be cited. There is no requirement to cite these works unless the editor has indicated otherwise.

Please review your reference list to ensure that it is complete and correct. If you have cited papers that have been retracted, please include the rationale for doing so in the manuscript text, or remove these references and replace them with relevant current references. Any changes to the reference list should be mentioned in the rebuttal letter that accompanies your revised manuscript. If you need to cite a retracted article, indicate the article’s retracted status in the References list and also include a citation and full reference for the retraction notice.

Additional Editor Comments (if provided):

[Note: HTML markup is below. Please do not edit.]

Reviewers' comments:

Reviewer's Responses to Questions

**Comments to the Author**

1. Does this manuscript meet PLOS Global Public Health’s publication criteria?

Reviewer #1: Yes

Reviewer #2: Yes

Reviewer #3: Yes

**********

2. Has the statistical analysis been performed appropriately and rigorously?

Reviewer #1: Yes

Reviewer #2: Yes

Reviewer #3: Yes

**********

3. Have the authors made all data underlying the findings in their manuscript fully available (please refer to the Data Availability Statement at the start of the manuscript PDF file)?

The PLOS Data policy

Reviewer #1: No

Reviewer #2: Yes

Reviewer #3: No

**********

4. Is the manuscript presented in an intelligible fashion and written in standard English?

Reviewer #1: Yes

Reviewer #2: Yes

Reviewer #3: Yes

**********

Reviewer #1: Overall comment

This is a well-conducted and important study addressing a key evidence gap on G6PD deficiency in a malaria-endemic setting in Latin America.

However, the manuscript would benefit from improvements in the Discussion section, which is currently difficult to follow.

Major Comments

1. Abstract: clarify G6PD methodology

The abstract would benefit from briefly specifying:

• which G6PD test was used, and

• how deficient, intermediate, and normal activity were defined

2. Terminology: “standard primaquine” vs “low-dose primaquine”

In the Introduction, the manuscript refers to “standard-dose primaquine.” Given that WHO guidelines now recommend high-dose primaquine in many settings, the term “standard” is ambiguous and potentially outdated. It would be clearer and more consistent to refer to this regimen throughout as “low-dose primaquine.”

3. Methods: G6PD testing

The section describing G6PD testing raises several points that require clarification:

• The manuscript describes repeat testing and notes that participants with Hb <7 g/dL should not have G6PD results interpreted.

• However, it is not clear:

o how repeat test results were handled in the analysis

o whether participants with Hb <7 g/dL were excluded, and if so, from which analyses

This information should be clearly stated in the Methods, rather than only becoming apparent later in the Results.

4. Reliability of self-reported adverse events

I am a bit doubtful if this section adds much and would be inclined to remove from the manuscript. Self reported events are notoriously difficult to interpret for recall bias etc. At a minimum it would be goo to add over which timeframe these events were recalled. Its not clear how this data supports any of the main messages in the manuscript.

5. Discussion: structure and readability

The Discussion is currently difficult to follow it feels disjointed from the rest of the manuscript. It would benefit from tightening overall to avoid repetition.

The first paragraph largely repeats numerical results, rather than providing a clear interpretative summary.

It is a bit confusing to jump between epidemiology / prevalence comparisons in the second paragraph to test inherent issues in the third paragraph back to prevalence among indigenous populations in the 4th paragraph. Perhaps it would make it easier on the reader to talk about prevalence findings first, then about measurement and classification issues.

Similarly the key discussion section on relationship of G6PD and malaria are difficult to read (paragraph 5, 6 7 and 8) and could be tighter.

6. Interpretation of policy implications (currently paragraph 10 of the discussion and conclusions)

In several places, the manuscript refers to implications for policy, but these are often vague or rather supportive of current guidance than new.

For example:

• 532 and 571: The suggestion that findings support the need for G6PD testing prior to high-dose primaquine or tafenoquine is not novel—this is already established WHO guidance. The presented data may support or reinforce this approach, but do not introduce a new policy direction.

Towards the end of the Discussion, the manuscript appears to suggest that approaches to radical cure and MDA might differ for the indigenous population. Are the authors suggesting that in these populations high dose primaquine or tafenoquine could be used without testing?

Minor

Introduction

• Line 99: missing closing bracket

• Line 110: missing space

Methods

• Line 137: add “febrile” for consistency with terminology used elsewhere

Results

• Line 354: “primaquine” is abbreviated as “PQ” without prior definition; ensure consistency throughout

• In line 474, the manuscript refers to a “dose-dependent effect” in relation to G6PD activity.

• This terminology is not appropriate in this context, as G6PD activity is a biological measure, not an administered exposure

Reviewer #2: The manuscript is well written and provides a useful piece of evidence on prevalence of G6PD deficiency in different populations of Colombia.

I only have minor comments and suggestions to improve the manuscript.

Throughout the text it is mentioned few times that G6PD activity might increase during a malaria episode; while this is expected and is partially explained by parasite-induced haemolysis, in P. vivax bone marrow suppression might counterbalance this phenomenon. At the moment, only few data have been collected properly on this issue so the statements in the text should be revised.

Table 2: prevalence of G6PDd in the indigenous group should not be reported as NA but as 0/N (where N is the total number of indigenous participants and females and males)

Table 2: the WHO classification is not meant to be used as a classification of individual activities; therefore, only the categorization in % of activity should be used without mention of WHO classification. The citation (26) used is not appropriate and should be removed. For more info on the recent WHO classification and its use on classification of G6PD variants, please see this link New WHO classification of genetic variants causing G6PD deficiency - PMC

Line 384 Can the authors explain why they choose to use normal AND intermediate as reference group?

When analysing G6PD activity in malaria patients and healthy population, I think it would make more sense to stick to the comparison of prevalence of different G6PD phenotypes in malaria cases vs control (healthy subjects). Using changes in G6PD activity levels might not be appropriate because the Biosensor is not a fully quantitative test, the level of G6PD activity and Hb are influenced by duration of infection and parasitemia which were factors not analysed.

The discussion is very long and could really be improved by removing information already given in the introduction and by streamlining the text by focusing on the main findings.

Reviewer #3: Thanks to the Editor and authors for the opportunity to review this interesting manuscript. Zuluaga-Idarraga et al investigate the prevalence of G6PD deficiency in the majority Afro-Columbian and minority Emberá dobida indigenous population in Quibdó, Colombia, as well as the association between G6PD activity and Plasmodium spp infection by PCR. G6PD deficiency was observed to be common among the predominantly Afro‑Colombian general population but absent in the indigenous group, which had the highest P. vivax burden.

Overall, the manuscript is clearly written, surveys and testing approaches are methodologically appropriate and the study provides relevant insights for guiding the targeted implementation of P. vivax radical cure. The analyses are appropriate and the findings are clearly presented, thoughtfully discussed and limitations noted.

Several minor revisions are suggested to improve clarity and interpretation of the study and it’s findings. I recommend acceptance pending the authors addressing these minor revisions.

1 - The details of the quantitative G6PD test used should be provided in the abstract.

2 - The cutoffs used to classify individuals as deficient, intermediate and normal should be provided in the abstract.

3 – Line 42-42 of abstract, should indicate that “The relationships between G6PD status, ethnicity and the risk of Plasmodium infection was evaluated.”

4 – Line 74 – please clarify that G6PDd is important in determining the safety of radical cure for P. vivax rather for radical cure itself.

5 – Table 1 provides data on consanguinity between parents, however there are no methodological details provided as to how genetic relatedness was determined.

6 - The authors need to clarify how they are making the full dataset available as per journal requirements. The data availability statement indicates that all data are provided within the manuscript or supplementary information files, however this only includes summary statistics.

**********

what does this mean?). If published, this will include your full peer review and any attached files.

**Do you want your identity to be public for this peer review?**  If you choose “no”, your identity will remain anonymous but your review may still be made public.

For information about this choice, including consent withdrawal, please see our Privacy Policy

Reviewer #1: No

Reviewer #2: No

Reviewer #3: No

**********

[NOTE: If reviewer comments were submitted as an attachment file, they will be attached to this email and accessible via the submission site. Please log into your account, locate the manuscript record, and check for the action link "View Attachments". If this link does not appear, there are no attachment files.]

# Author comment: Author response to Decision Letter 1

**DOI:** 10.1371/journal.pgph.0007460.r003

24 Jul 2026

Attachment Submitted filename: Response to Reviewers.docx

# Editor report: Decision Letter 1

**DOI:** 10.1371/journal.pgph.0007460.r004

**Authors:** Shazia Ruybal-Pesántez

13 Aug 2026

PGPH-D-26-00377R1

Prevalence of glucose-6-phosphate dehydrogenase (G6PD) deficiency in a malaria endemic region of Colombia: implications for radical cure of Plasmodium vivax

PLOS Global Public Health

Dear Dr. Zuluaga-Idarraga,

Thank you for submitting your manuscript to PLOS Global Public Health. After careful consideration, we feel that it has merit but does not fully meet PLOS Global Public Health’s publication criteria as it currently stands due to some grammatical and typographical errors. Therefore, we invite you to submit a revised version of the manuscript that addresses the points raised during the review process.

Please submit your revised manuscript by Sep 12 2026 11:59PM. If you will need more time than this to complete your revisions, please reply to this message or contact the journal office at globalpubhealth@plos.org. When you're ready to submit your revision, log on to https://www.editorialmanager.com/pgph/ and select the 'Submissions Needing Revision' folder to locate your manuscript file.

Please include the following items when submitting your revised manuscript:

- A letter that responds to each point raised by the editor and reviewer(s). You should upload this letter as a separate file labeled 'Response to Reviewers'.
- A marked-up copy of your manuscript that highlights changes made to the original version. You should upload this as a separate file labeled 'Revised Manuscript with Track Changes'.
- An unmarked version of your revised paper without tracked changes. You should upload this as a separate file labeled 'Manuscript'.

Guidelines for resubmitting your figure files are available below the reviewer comments at the end of this letter.

As the corresponding author, your ORCID iD is verified in the submission system and will appear in the published article. PLOS supports the use of ORCID, and we encourage all coauthors to register for an ORCID iD and use it as well. Please encourage your coauthors to verify their ORCID iD within the submission system before final acceptance, as unverified ORCID iDs will not appear in the published article. *Only*  the individual author can complete the verification step; PLOS staff *cannot*  verify ORCID iDs on behalf of authors.

We look forward to receiving your revised manuscript.

Kind regards,

Shazia Ruybal-Pesántez

Academic Editor

PLOS Global Public Health

**Journal Requirements:**

If the reviewer comments include a recommendation to cite specific previously published works, please review and evaluate these publications to determine whether they are relevant and should be cited. There is no requirement to cite these works unless the editor has indicated otherwise.

Please review your reference list to ensure that it is complete and correct. If you have cited papers that have been retracted, please include the rationale for doing so in the manuscript text, or remove these references and replace them with relevant current references. Any changes to the reference list should be mentioned in the rebuttal letter that accompanies your revised manuscript. If you need to cite a retracted article, indicate the article’s retracted status in the References list and also include a citation and full reference for the retraction notice.

**Additional Editor Comments (if provided):**

The reviewers comments were addressed, however there are some grammatical and typographical errors that remain in this revised version. Please thoroughly check for any typos or grammatical errors throughout the manuscript, some examples below (these are non-exhaustive, please revise carefully):

L37: Plasmodium should be fully written upon first mention and then abbreviated

L52: "respectively" repeated

L149: outpatients febrile -> febrile outpatients

L442: Value were not estimated because some categories were not contained G6PDd cases -> Values were not estimated because some categories did not contain G6PDd cases.

L481: statistically difference -> statistically different

L493: vivax -> P. vivax

**Reviewers' comments:**

[NOTE: If reviewer comments were submitted as an attachment file, they will be attached to this email and accessible via the submission site. Please log into your account, locate the manuscript record, and check for the action link "View Attachments". If this link does not appear, there are no attachment files.]

**Figure Resubmissions:**

# Author comment: Author response to Decision Letter 2

**DOI:** 10.1371/journal.pgph.0007460.r005

10 Sep 2026

Attachment Submitted filename: RESPON~1.DOC

# Editor report: Decision Letter 2

**DOI:** 10.1371/journal.pgph.0007460.r006

**Authors:** Shazia Ruybal-Pesántez

28 Sep 2026

Prevalence of glucose-6-phosphate dehydrogenase (G6PD) deficiency in a malaria endemic region of Colombia: implications for radical cure of Plasmodium vivax

PGPH-D-26-00377R2

Dear Dr Zuluaga-Idarraga,

We are pleased to inform you that your manuscript 'Prevalence of glucose-6-phosphate dehydrogenase (G6PD) deficiency in a malaria endemic region of Colombia: implications for radical cure of Plasmodium vivax' has been provisionally accepted for publication in PLOS Global Public Health.

Before your manuscript can be formally accepted you will need to complete some formatting changes, which you will receive in a follow up email. A member of our team will be in touch with a set of requests.

Please note that your manuscript will not be scheduled for publication until you have made the required changes, so a swift response is appreciated.

IMPORTANT: The editorial review process is now complete. PLOS will only permit corrections to spelling, formatting or significant scientific errors from this point onwards. Requests for major changes, or any which affect the scientific understanding of your work, will cause delays to the publication date of your manuscript.

If your institution or institutions have a press office, please notify them about your upcoming paper to help maximize its impact. If they'll be preparing press materials, please inform our press team as soon as possible -- no later than 48 hours after receiving the formal acceptance. Your manuscript will remain under strict press embargo until 2 pm Eastern Time on the date of publication. For more information, please contact globalpubhealth@plos.org.

Thank you again for supporting Open Access publishing; we are looking forward to publishing your work in PLOS Global Public Health.

Best regards,

Shazia Ruybal-Pesántez

Academic Editor

PLOS Global Public Health

***********************************************************

Reviewer Comments (if any, and for reference):
